# The potential role of sex related differences of the microbiome and its impact on calcific aortic stenosis: a narrative review

**DOI:** 10.1186/s13293-026-00932-7

**Published:** 2026-05-29

**Authors:** Sarah Atighetchi, Thomas Pilgrim, Yilmaz Bahtiyar, Gaspard Suc, Marina Urena-Alcazar, Yvonne Döring, Caroline Chong-Nguyen

**Affiliations:** 1 Department of Cardiology, Bern University Hospital, Inselspital, University of Bern, Freiburgstrasse 20, Bern, CH-3010 Switzerland; 2 Department of General Internal Medicine, Inselspital, Bern University Hospital, University of Bern, Bern, Switzerland; 3 Institute of Primary Health Care (BIHAM), University of Bern, Bern, Switzerland; 4 Department of Visceral Surgery and Medicine, Bern University Hospital, University of Bern, Bern, 3010 Switzerland; 5 Department for Biomedical Research, Maurice Müller Laboratories, University of Bern, Bern, 3008 Switzerland; 6 Department of Cardiology, Bichat Hospital, Assistance Publique-Hôpitaux de Paris, Paris, France; 7 UMRS 1148, INSERM, Université Paris Cité, Paris, 75018 France; 8 Department of Angiology, Swiss Cardiovascular Center, Inselspital, Bern University Hospital, University of Bern, Bern, Switzerland; 9 Institute for Cardiovascular Prevention (IPEK), Ludwig-Maximilians-Universität, Munich, Germany; 10 Department of Cardiology, Biel/Bienne Hospital, Bern, Switzerland; 11 Department for Biomedical Research, University of Bern, Bern, 3008 Switzerland; 12 Metabolism Inflammation Digital Health Osteology (MIDHOS), Faculty of Medicine, University of Bern, Bern, Switzerland

**Keywords:** Gut microbiota, Metabolomics, Sex difference, Calcific aortic stenosis

## Abstract

Aortic stenosis (AS) presents with distinct sex-related differences in clinical manifestation, pathophysiology, and response to treatment. Women typically present at an older age, with greater frailty, more pronounced symptoms, and paradoxical low-flow AS, often associated with concentric left ventricular remodeling and fibrotic valve changes. In contrast, men show a predominance of calcific AS, eccentric remodeling, and more extensive aortic valve calcification. These differences are not solely anatomical or hemodynamic; they extend to molecular pathways and emerging contributors such as the gut microbiota.

Recent evidence suggests that gut microbiota composition and its metabolites, particularly trimethylamine-N-oxide (TMAO) and indoxyl sulfate (IS), play a sex-specific role in AS pathogenesis. Women generally exhibit a more diverse and cardioprotective microbiota profile, shaped by estrogen and dietary habits, that might explain lower levels of pro-calcific metabolites and a fibrotic valve phenotype. Conversely, men tend to have higher TMAO and IS levels, driven by a *Firmicutes*-enriched microbiota and androgenic modulation, which might promote calcification and inflammatory signaling in the aortic valve.

This review integrates current knowledge on sex-related differences in AS, spanning clinical patterns, valvular remodeling, cellular and molecular signaling, and gut–heart interactions, to propose a hypothesis-driven framework on how gut microbiota may contribute to sex-specific differences in AS.

## Abstract (plain language summary)

Aortic stenosis (AS) affects men and women differently. Women are usually older when the disease is diagnosed, often feel more symptoms, and tend to have a form of the disease with less blood flow and more stiffening of the heart muscle. Their heart valves are more fibrotic (scar-like). Men, on the other hand, more often develop heavily calcified (hardened) valves and a different pattern of heart enlargement.

These differences are not just about heart structure or blood flow — they may also be linked to deeper biological processes, including the gut microbiome (the bacteria living in our intestines).

Recent research suggests that gut bacteria and the substances they produce can influence how AS develops, and this may differ between men and women. Women tend to have a more diverse and protective gut microbiome, partly influenced by hormones like estrogen and diet. This may lead to lower levels of harmful substances and more fibrotic (less calcified) valve changes. Men, in contrast, often have higher levels of certain metabolites (such as TMAO and indoxyl sulfate) that have been shown in the literature to be linked to inflammation and valve calcification.

This review brings together current knowledge on how sex differences, heart changes, biological pathways, and the gut microbiome interact in AS to propose a hypothesis-driven framework linking gut microbiota and its metabolites to sex-specific differences in AS pathophysiology.

## Introduction

Aortic stenosis (AS) is the most prevalent primary valvular heart disease in Europe and Northern America, with a rising prevalence driven by population ageing [1]. Approximately 2% of individuals aged ≥ 65 years are affected, increasing to 4.6% in those aged ≥ 75 years [2, 3]. Anatomically, the human aortic valve is a thin, avascular trileaflet structure composed of valve endothelial cells (VECs) on the surface and three internal layers, the fibrosa, spongiosa, and ventricularis, each with distinct extracellular matrix (ECM) components and mechanical roles. VECs maintain homeostasis, while valve interstitial cells (VICs) produce structural proteins. The fibrosa bears mechanical stress, the spongiosa provides cushioning, and the ventricularis enhances elasticity. Degenerative processes represent the leading cause, with aortic valve calcification as the central mechanism underlying valvular stiffening and obstruction [4–7]. Once considered a passive consequence of ageing, AS is now recognized as an active process sharing pathophysiological features with atherosclerosis, including lipid infiltration, endothelial injury, inflammation, extracellular matrix remodelling, fibrosis, and progressive calcification [8–13].

Sex-related differences have been consistently reported in AS. Men account for ~ 70% of cases [9, 14] and typically present with a higher calcification burden [15, 16]. In contrast, women often develop more severe haemodynamic stenosis for the same aortic valve calcification burden, likely due to a higher proportion of valvular fibrosis [15–21]. Distinct risk profiles have also been identified: dyslipidemia is a stronger driver of calcification progression in men, whereas hypertension predominates in women [22]. These differences extend to procedural outcomes, with women showing improved long-term survival after transcatheter aortic valve implantation (TAVI) [23] compared with men, despite higher early complication rates [24–32].

Beyond traditional cardiovascular risk factors, emerging evidence highlights the gut microbiota as a potential contributor to AS pathogenesis. The gut microbiota, comprising trillions of microorganisms whose composition is shaped by age, diet, comorbidities, and sex hormones [33–40], shows sex-specific patterns with implications for immune regulation, metabolism, and cardiovascular risk [41, 42]. Dysbiosis can promote inflammatory activation, alter lipid metabolism, and increase production of pro-atherogenic metabolites such as trimethylamine N-oxide (TMAO), which are implicated in atherosclerosis [43, 44] and may also contribute to AS. Importantly, accumulating evidence indicates that microbiota composition and gut-derived metabolites differ between men and women, potentially influencing inflammatory, fibrotic, and calcific pathways in valve disease [43]. Given the overlap between mechanisms of atherosclerosis and AS, sex-related alterations in the gut microbiome may help explain differences in AS presentation, progression, and outcomes—though this field remains largely unexplored and warrants further investigation.

## Methods

### Study design

This narrative review was conducted to summarize current evidence on sex differences in AS, with a specific focus on the potential role of gut microbiota and microbiota-derived metabolites. Given the emerging and heterogeneous nature of the field, a systematic review or meta-analysis was not feasible. Instead, a structured narrative synthesis approach was used to integrate evidence across clinical, experimental, and translational studies.

Although not formally registered, the review was conducted in accordance with the general principles of PRISMA-ScR to ensure transparency in study identification, selection, and synthesis of the literature [45].

### Literature search strategy

A structured literature search was performed to identify relevant publications. Searches were conducted in **PubMed and EMBASE**, covering articles published in **English** from 1st December 2015 up to **November 2025**.

Given the absence of a dedicated body of literature directly addressing sex differences in gut microbiota and aortic stenosis, a multi-step search strategy was applied using three complementary search blocks:

1. **“Aortic stenosis” AND “gut microbiota” AND “sex”**.

#### Keywords

(Aortic Stenosis[Mesh] OR aortic stenosis OR calcific aortic stenosis)

AND.

(Gastrointestinal Microbiome[Mesh] OR gut microbiota OR gut microbiome OR microbiome OR TMAO OR trimethylamine N-oxide OR indoxyl sulfate OR tryptophan metabolism)

AND.

(Sex Factors[Mesh] OR Sex Differences[Mesh] OR sex differences OR sex-specific OR gender differences OR male female OR men women OR estrogen OR testosterone OR sex hormones)

2. **“Aortic stenosis” AND “sex differences”**.

#### Keywords

((“Aortic Valve Stenosis“[Mesh] OR “Aortic Stenosis“[Mesh] OR “Aortic Valve Calcification“[Mesh] OR “Aortic Valve Fibrosis“[Mesh] OR (aortic AND (stenos* OR sclerosis OR calcif* OR fibros*))) AND (“Sex Factors“[Mesh] OR “Sex Characteristics“[Mesh] OR “Sex Distribution“[Mesh] OR “Sex Differences“[Mesh] OR (sex* OR gender difference* OR “male vs female” OR “men vs women”) OR (hormone* AND (estrogen OR androgen OR testosterone OR progesterone)) OR (menopaus* OR postmenopaus* OR sex-specific OR gender-specific)) AND (“TAVI” OR “Transcatheter Aortic Valve Replacement“[Mesh] OR “TAVR” OR “SAVR” OR “Aortic Valve Replacement” OR “Surgical Aortic Valve Replacement” OR “SAVR outcomes” OR “TAVI outcomes” OR “transcatheter aortic valve implantation” OR “surgical outcomes” OR “aortic valve surgery”)) AND (“sex-based outcomes” OR “gender-specific outcomes” OR “sex-related differences” OR “gender-related differences” OR “female-specific outcomes” OR “male-specific outcomes” OR “prognostic differences by sex” OR “sex-stratified analysis” OR “sex hormones and valve disease” OR “impact of gender on outcomes” OR “sex disparities in TAVI” OR “gender disparities in SAVR” OR “influence of sex on mortality” OR “sex differences in procedural outcomes”).

3. **“Gut microbiota” AND “sex differences”**.

#### Keywords

(“Gastrointestinal Microbiome“[Mesh] OR “Intestinal Microbiome“[Mesh] OR “Microbiota“[Mesh] OR (gut microbiota OR gut microbiome OR intestinal microbiota OR intestinal microbiome)) AND (“Sex Differences“[Mesh] OR “Sex Factors“[Mesh] OR “Sex Characteristics“[Mesh] OR (sex difference* OR gender difference* OR “male vs female” OR “men vs women” OR sex-specific OR gender-specific)) AND (hormone* OR estrogen OR androgen OR testosterone OR progesterone OR menopaus* OR postmenopaus* OR “microbiota composition” OR “gut microbiota diversity” OR “microbiome sex dimorphism” OR “microbiota and hormones”).

Additional relevant articles were identified through manual screening of reference lists from included studies and key review articles.

### Eligibility criteria

We included original research articles, observational studies (cross-sectional, case-control, retrospective, and prospective cohort studies), interventional studies reporting baseline data, and experimental studies when relevant to mechanistic interpretation. Narrative reviews and mechanistic studies were used selectively to support biological interpretation but were not considered primary evidence.

Eligible studies involved:

- adult human Population (≥ 18 years), or.
- preclinical models relevant to AS, sex differences, or gut microbiota-derived metabolites.

Studies were included if they reported at least one of the following:

- gut microbiota composition (taxonomic or diversity-based analyses).
- microbiota-derived metabolites (e.g., TMAO, tryptophan derivatives, bile acids and SCFAs).
- sex-stratified analyses or sex-specific biological effects.

Only peer-reviewed articles published in English were included.

### Study selection

Titles and abstracts identified through the search strategy were screened for relevance. Full-text articles were then assessed for eligibility based on predefined inclusion criteria. Reference lists of included studies were manually screened to identify additional relevant publications.

Given the exploratory nature of this review, study selection prioritized biological relevance over methodological homogeneity.

### Data synthesis

Due to the heterogeneity of study designs, populations, and outcomes, a quantitative synthesis was not performed. Instead, findings were summarized narratively.

Data extraction focused on:

- study design and population characteristics.
- microbiota composition (taxonomic differences, diversity indices).
- metabolite profiles (particularly TMAO, tryptophan derivatives, bile acids and SCFAs).
- sex-specific differences in biological or clinical outcomes.
- mechanistic pathways linking microbiota, metabolites, and valvular remodeling.

### Methodological considerations

Given the lack of studies directly addressing the combined interaction between sex, gut microbiota, and AS, this review necessarily integrates indirect evidence from multiple related fields, including cardiovascular disease, metabolic disease, and experimental valve biology.

As such, conclusions are hypothesis-generating and intended to provide a conceptual framework for future experimental and clinical research rather than definitive causal inference.

### Definition of sex

In this review, sex refers to biological attributes distinguishing male and female individuals, including chromosomal, hormonal, and physiological characteristics that may influence cardiovascular and microbial phenotypes.

## Results and discussion

### Literature search

The systematic literature search identified a total of 960 potentially relevant publications across three distinct research axes.

For the first axis focusing on Aortic Stenosis (AS) and sex differences, 368 studies were initially identified. Following a primary screening for clinical relevance and pathophysiological mechanisms, 91 studies were selected for full-text evaluation, of which 36 studies met the eligibility criteria and were included in the final synthesis.

Regarding the second axis on gut microbiota and sex differences, the search yielded 573 results. After screening 80 publications for relevance to hormonal regulation and microbial metabolites, 48 studies were retained for the final analysis.

**Fig. 1:**
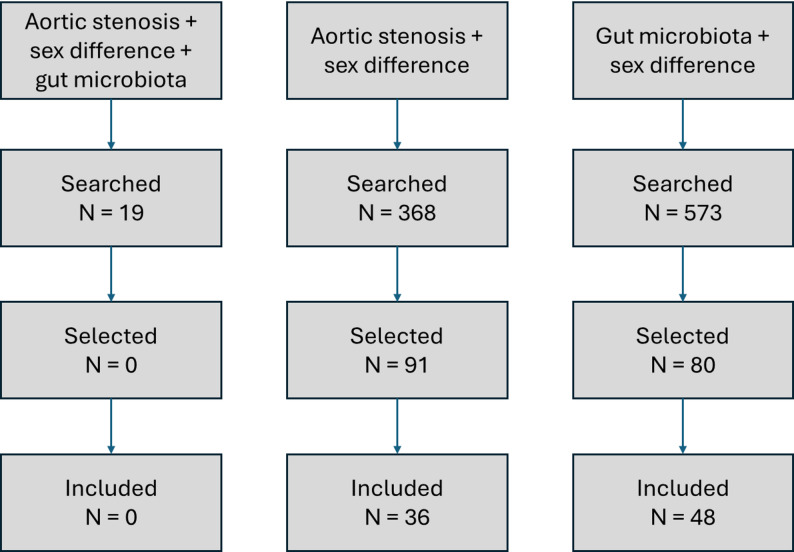
Study Selection Flow Diagram. This diagram illustrates the multi-step identification and screening process used to synthesize current evidence. The literature search was categorized into three distinct thematic axes: (**A**) Aortic Stenosis (AS) and sex differences, (**B**) Gut microbiota and sex differences, and (**C**) The intersection of Aortic Stenosis, sex, and gut microbiota

Finally, while 19 publications were initially identified at the triple intersection of aortic stenosis, sex, and gut microbiota, a detailed assessment revealed that none met the specific inclusion criteria for a direct, integrated analysis of all three components (Fig. 1).

### Sex differences in gut microbiota composition

**Fig. 2:**
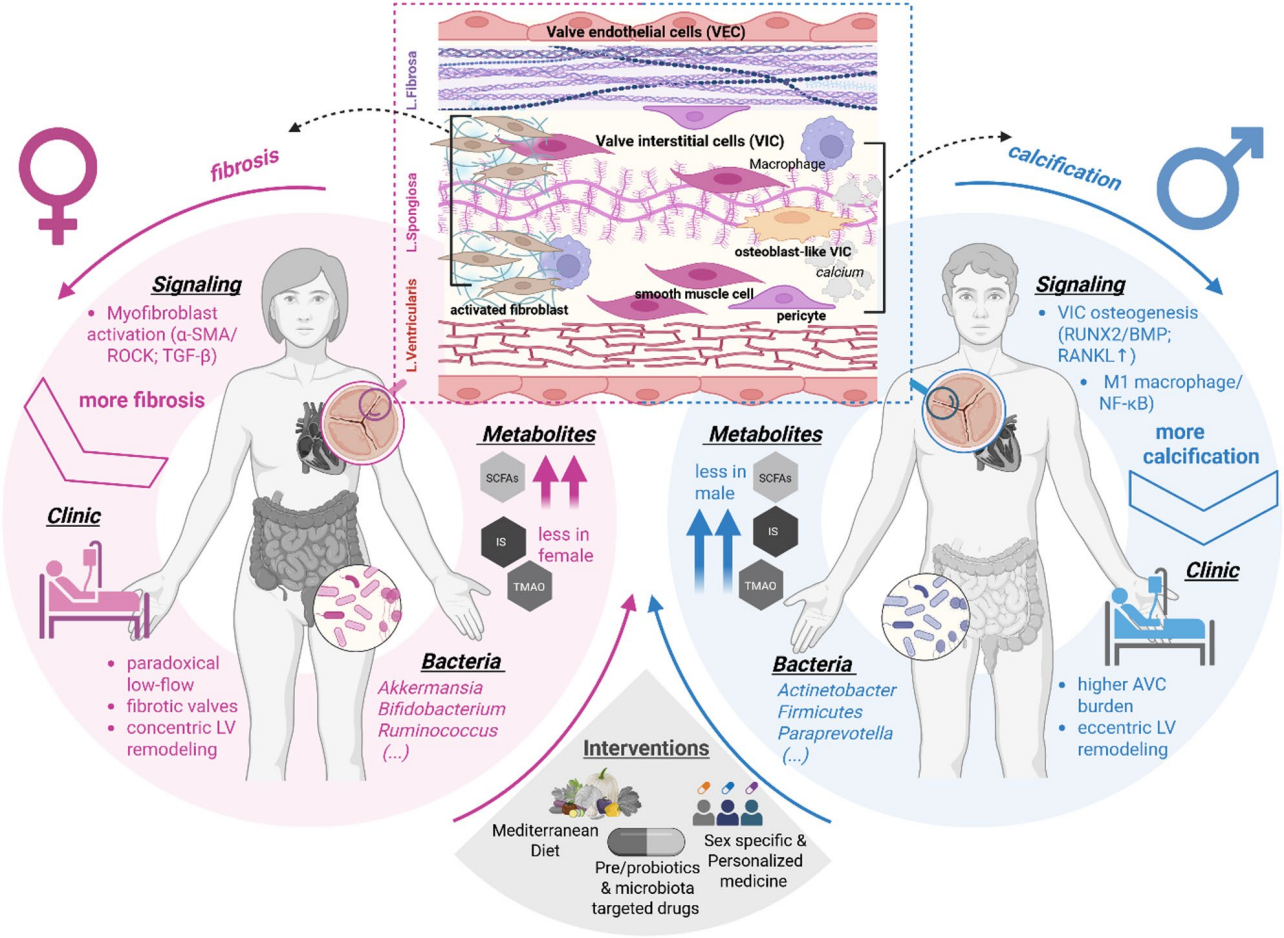
Potential sex-specific interplay between gut microbiota, metabolites, valve cell biology, and clinical phenotypes in aortic stenosis (AS). Schematic illustration of a hypothesis-driven framework integrating sex differences in gut microbiota composition, metabolite profiles, valve biology, and clinical phenotypes in aortic stenosis. Female patients (left, pink): Gut microbiota enriched in *Akkermansia*,* Bifidobacterium*, and *Ruminococcus* has been associated with increased production of short-chain fatty acids (SCFAs) and relatively lower circulating levels of trimethylamine-N-oxide (TMAO) and indoxyl sulfate (IS). Reduced exposure to pro-inflammatory and pro-calcific metabolites, together with estrogen-related effects, may be associated with enhanced myofibroblast activation (α-SMA, ROCK, TGF-β signaling) and a predominantly fibrotic pattern of valvular remodeling. This phenotype is clinically observed in association with paradoxical low-flow AS and concentric left ventricular (LV) remodeling. Male patients (right, blue): Microbiota enriched in *Actinobacter*, *Firmicutes*, and *Paraprevotella* are associated with higher circulating TMAO and IS. These metabolites are described to promote VIC osteogenesis (RUNX2, BMP, RANKL↑) and immune infiltration (M1 macrophages, NF-κB activation), driving calcification. We hypothesize that in males with AS, if the same gut microbiota composition and metabolomic profile are observed, this can be associated with a greater burden of aortic valve calcification and a tendency toward eccentric LV remodeling. Valve cell biology (center inset): Valve interstitial cells (VICs) represent a heterogeneous population including activated fibroblasts, smooth muscle-like cells, osteoblast-like VICs, and pericytes. Inflammatory cells such as macrophages contribute to both fibrotic and calcific remodeling. As described in the literature, women show a predominance of fibrotic remodeling, whereas men show more calcific nodule formation. Within the proposed framework, gut microbiota–derived metabolites such as TMAO, indoxyl sulfate, and SCFAs may represent upstream modulators of VIC phenotypic switching, potentially influencing the balance between fibrotic and osteogenic activation states in a sex-dependent manner. Interventions (bottom): Potential translational approaches may include dietary modulation (e.g., Mediterranean diet patterns), prebiotic/probiotic strategies, and microbiota-targeted interventions. These concepts remain exploratory and require further validation in the context of sex-specific AS pathophysiology. *This figure was made with Biorender.com*

Recent large-scale studies [41] using advanced sequencing techniques have revealed subtle but consistent differences in the gut microbiota composition between healthy men and women [46, 47]. Overall, healthy women tend to have greater microbial α-diversity and a higher relative abundance of beneficial taxa such as *Akkermansia muciniphila*,* Bifidobacterium*,* Ruminococcus*, and members of *Lactobacillales* and *Streptococcaceae*. In contrast, healthy men are more often enriched in *Prevotella*,* Bacteroides*,* Veillonella*,* Methanobrevibacter*, and *Coprococcus catus* [41] (Fig. 2). These sex-based differences are modest in magnitude but reproducible across cohorts in Europe, the USA, and Asia, with some variation depending on geographical and dietary contexts [47].

Sex steroid hormones are recognized as key factors associated with gut microbiota composition, contributing to sex-based differences in microbial diversity and function. Estrogens, progesterone, and testosterone not only influence systemic metabolism but also directly modulate the gut microbiome by altering the intestinal environment and microbial gene expression [48]. Studies have shown that estrogen-rich environments promote the growth of certain bacterial species while inhibiting others, leading to distinct microbial profiles in males and females [49].

Testosterone also affects microbial composition, with higher levels in males favoring bacteria linked to reduced inflammation, whereas lower testosterone levels in females are associated with a microbiota composition that may predispose to autoimmune and metabolic diseases [50]. Sex hormones shape gut microbiota by influencing gut permeability, immune responses, and bile acid metabolism. Estrogen regulates gut motility and mucus production, creating a unique environment for bacterial populations. This interaction is bidirectional, as gut microbes also modulate sex hormone metabolism. Non-steroidal hormones like insulin, cortisol, and growth hormone further impact microbial composition by affecting immune function and energy metabolism [51, 52].

In men, high testosterone levels are associated with an increased abundance of *Acinetobacter* (*Proteobacteria*), *Dorea* (*Firmicutes*), *Ruminococcus* (*Firmicutes*), and *Megamonas* (*Firmicutes*) [53]. In contrast, women with high estradiol levels exhibit greater proportions of *Slackia* (*Actinobacteria*) and *Butyricimonas* (*Bacteroidetes*), along with an overall increase in the *Bacteroidetes* phylum and a decrease in *Firmicutes*. Furthermore, *Paraprevotella* (*Bacteroidetes*) exhibits a positive correlation with testosterone and a negative correlation with estradiol, suggesting a sex-dependent microbial shift [54].

Diet plays a crucial role in shaping gut microbiota composition. The Western diet (WD), rich in saturated fats, refined sugars, and processed foods, is associated with lower microbial diversity, increased pro-inflammatory species, and reduced beneficial bacteria. In contrast, the Mediterranean diet (MD), characterized by high consumption of fruits, vegetables, whole grains, and healthy fats, promotes microbial diversity and enhances anti-inflammatory and beneficial bacteria. Men and women tend to have different dietary habits [55], which influence gut microbiota. Women typically consume more fruits, vegetables, and fiber, which has been associated with a higher abundance of beneficial bacteria such as *Bifidobacterium* and Lactobacillus. In contrast, men often consume more protein and fat, favoring the growth of *Bacteroides* species such as *Prevotella* [56].

These observations represent primarily data derived from healthy populations and constitute indirect evidence of sex-related microbial differences, rather than disease-specific findings in AS.

Emerging evidence suggests that the gut microbiota may be involved in cardiovascular disease processes and has been hypothesized to contribute to the pathophysiology of AS, potentially influencing disease progression and sex-related differences through mechanisms such as inflammation [43].

## Sex difference in gut microbiota derived metabolites

The gut microbiota may influence the sex differences observed in AS by interacting with sex hormones, which affect both the composition of the microbiota and the progression of the disease. Estrogen, which is more prominent in women, has protective effects on cardiovascular health [57] and may modulate the gut microbiota in ways that reduce the production of harmful metabolites, such as TMAO. These metabolites are linked to the progression of AS by promoting fibrosis and calcification in the aortic valve [58, 59]. However, whether sex-related differences in gut microbiota and metabolite profiles directly contribute to the divergent AS phenotypes observed in men and women remains to be established.

### TMAO

Evidence derived from experimental and clinical studies in aortic stenosis indicates that TMAO is involved in valvular remodeling processes.

TMAO levels are generally lower in women [60, 61], which could lower the calcification load, as studies have demonstrated that elevated TMAO levels in both animal and cell models exacerbate the calcification of aortic valves. This elevated TMAO promotes calcification in male VICs by enhancing osteogenic differentiation through signaling pathways such as PI3K/Akt and STAT1-ERK-HIF1α, which are more active in male VICs [62, 63].

Men with calcific AS exhibit greater macrophage infiltration and activation in the aortic valve than women, which may be partly explained by higher circulating levels of TMAO. TMAO promotes M1 macrophage polarization and activates the NF-κB inflammatory pathway, enhancing the pro-inflammatory environment in the aortic valve. These activated macrophages, in turn, stimulate inflammatory responses in aortic VICs, possibly contributing to disease progression. In vivo studies support this mechanism, showing that loss of negative regulators of NF-κB accelerates valve lesion development in the presence of high TMAO levels [64]. This mechanism may represent a relevant area for further investigation in relation to the sex-specific inflammatory and calcific phenotypes observed in male patients.

Recent studies have also highlighted the role of TMAO in promoting aortic valve fibrosis through the activation of endoplasmic reticulum (ER) stress pathways, specifically the PERK/ATF-4 and IRE-1α/XBP-1s signaling axes, both in vitro and in vivo [59]. These pathways are known to contribute to fibrotic processes, and TMAO-mediated activation of these signaling cascades may be relevant in the context of fibrotic remodeling observed in aortic valve disease.

Evidence from microbiota studies from other diseases provides insight into the regulation of TMAO production and its potential sex-related differences.

Certain members of the *Firmicutes* phylum, particularly bacteria from the *Lachnospiraceae* family, are known to be efficient producers of TMA, the precursor of TMAO. Men tend to have a higher relative abundance of these *Firmicutes*, which could lead to increased TMA production and consequently elevated TMAO levels after hepatic oxidation [60]. One keystone species that has been highlighted in AS is *Prevotella* [65], which is often found to be more abundant in male patients. Certain Prevotella-enriched microbiota profiles have been associated with enhanced conversion of dietary substrates such as choline and carnitine into trimethylamine (TMA), the precursor of TMAO [66]. In contrast, women’s gut microbiota is characterized by higher levels of Bifidobacterium and Akkermansia, which may counterbalance the pro-inflammatory effects of TMAO and be associated which may counterbalance pro-inflammatory effects of TMAO and be associated with a more fibrotic valvular profile [67, 67, 68].

Taken together, these observations support a hypothesis-generating framework linking microbiota-derived metabolites to sex-specific differences in aortic stenosis.

The differential response to gut microbiota-derived metabolites, such as TMAO, in men and women may help explain the sex-specific phenotypes of aortic valve disease. This difference in microbiota composition could partially explain the earlier onset and more severe progression of AS in men, as higher TMAO levels correlate with increased valve thickening and fibrosis in cell studies [59]. Sex-related differences in response to gut microbiota-derived metabolites, such as TMAO, represent a potential mechanism that warrants further investigation in the context of sex-specific aortic valve phenotypes.

Thus, the interplay between sex-specific microbial communities, metabolite profiles, hormonal regulation, ER stress signaling, and VIC biology likely underpins the distinct pathophysiological outcomes of AS observed in men and women. It is possible that the lower TMAO levels in women contribute to a slower progression of valve calcification: sex-related differences in circulating TMAO levels and hormonal milieu may influence the biological response to these metabolites, suggesting that VIC activation may not follow a simple dose-dependent or binary pattern but instead be differentially modulated in men and women. but this remains speculative and warrants further investigation to establish a clear causal relationship.

### Indoxyl sulfate (IS)

Evidence from experimental and translational studies indicates that IS is involved in valvular and vascular remodeling processes relevant to aortic stenosis. IS is a metabolite derived from the bacterial fermentation of tryptophan in the gut, which is then sulfonated in the liver. It is primarily produced by the gut microbiota and is involved in various pathological processes, including inflammation and fibrosis. IS is described to impair human aortic VECs by triggering inflammation, endothelial-to-mesenchymal transition, and calcification, partly through downregulation of integrin-linked kinases [69]. A recent study [70] showed that IS, especially when combined with phosphate, promotes calcification and upregulates the gene NKD2, a key mediator of pro-calcific and inflammatory responses. NKD2 knockdown reduces calcification and Interleukin-6 (IL-6) expression. In vivo, mice with IS supplementation or chronic kidney disease (CKD)-induced uremia showed worsened AS after valve injury, characterized by NKD2 expression and macrophage infiltration. IS also promotes the differentiation of monocytes into inflammatory subtypes and their adhesion to VECs.

Additional evidence from cardiovascular and renal diseases, as well as microbiota-related studies, provides insight into IS regulation and its systemic effects. In diseases outside of AS, such as chronic kidney disease, IS promotes pro-inflammatory macrophage activation. This effect is mediated via cellular uptake through OATP2B1 and activation of Notch signaling, contributing to vascular inflammation and atherosclerosis [71]. IS levels tend to be higher in men compared to women, particularly in diseases like coronary artery disease [72]. However, the reasons why men tend to have higher IS levels in coronary artery disease or chronic kidney disease, remain unclear, and there is no direct evidence in the literature linking these sex differences to AS specifically as well as no study assessing the level of IS in men and women in AS. This higher level of IS observed in men may be influenced by dietary patterns as men more often consume a WD rich in animal products, which alters gut microbiota composition and favors greater production of uremic toxins such as IS. By contrast, women are more likely to follow a MD, higher in plant-based foods, which is associated with microbiota profiles producing fewer harmful metabolites [55, 73, 74]. Estrogens, particularly estradiol, have additional protective effects against IS toxicity by modulating inflammatory responses and improving endothelial function, even though they do not directly lower IS levels [75].

These observations help supporting a hypothesis-generating framework linking IS, gut microbiota, and sex-specific differences in AS. Alongside with TMAO, IS shapes immune responses: in men, elevated levels drive M1 macrophage polarization and NF-κB activation, that could favor a pro-calcific inflammatory milieu, while in women, reduced exposure to these metabolites, coupled with short-chain fatty acids (SCFA)-mediated immune modulation from *Lactobacillales* and *Bifidobacterium*, that might support a less inflammatory, more fibrotic remodeling process.

Together, these differences, influenced by diet, hormonal status, and microbiota composition, may be associated with the distinct clinical patterns observed between sexes, with men tending to develop earlier and more severe calcific AS, and women more often presenting later with fibrotic valve phenotypes and paradoxical low-flow disease [76].

## Potential impact of sex and microbiota differences on clinical presentation of AS

Robust clinical and mechanistic evidence demonstrates marked sex-specific differences in the presentation, remodeling, and outcomes of AS. Women with AS tend to present at later stages of the disease, being older, more symptomatic, frailer, and having higher operative risks compared to men [77, 78]. They more often report exertional dizziness and dyspnea, and although they are less likely to have concomitant coronary artery or peripheral artery disease, they experience a greater symptom burden at similar AS severity compared to man [79]. Anatomically, women generally have smaller aortic valve areas and left ventricular (LV) outflow tracts, resulting in lower stroke volumes and a higher prevalence of paradoxical low-flow, low-gradient AS. Chronic pressure overload leads to concentric LV remodeling and hypertrophy in women, with reduced LV cavities, higher filling pressures, lower wall stress, and more pronounced diastolic dysfunction [80], whereas men typically develop dilated, eccentric LV remodeling [81]. At the valvular level, women tend to have less calcification but greater fibrosis. Despite these differences, women are frequently underdiagnosed, with disease severity underestimated and referral for aortic valve replacement (AVR) delayed. When treated, women derive greater long-term survival benefit from TAVI compared to men, although they experience higher 30-day vascular complications and bleeding [82]. In contrast, surgical AVR (SAVR) is technically more demanding in women and carries higher operative risk with worse outcomes and increased mortality, to the extent that female sex is considered a risk factor for SAVR [77]. 

The pathophysiology of AS shares similarities with atherosclerosis, involving endothelial damage, lipid accumulation, inflammation, fibrosis, and calcification [83]. Women with AS, even with similar hemodynamic severity to men, show less AV calcification, prompting the need for sex-specific thresholds in CT-based AS severity assessment [84] as their valves are described more fibrotic. Women develop severe aortic stenosis with lower calcification scores than men, as their disease is more often driven by valve fibrosis rather than calcification. In contrast, calcification predominates in men, resulting in higher calcium score thresholds. Therefore, sex-specific cut-off values are proposed, with lower AV calcium score thresholds defining severe AS in women [85, 86].

Gene expression differences, particularly in women, may explain enhanced valvular fibrosis, with activation of the myofibroblast pathway and X-chromosome-inactivation escape genes [87, 88]. In patients undergoing TAVI, women with higher AV calcium scores show increased mortality risks [89]. Women with AS often exhibit left ventricular concentric remodeling, whereas men tend to show eccentric remodeling. They also tend to have more diffuse myocardial fibrosis, which contributes to reduced left ventricular compliance, higher filling pressures, and advanced diastolic dysfunction [90]. This difference in myocardial remodeling may promote paradoxical low-flow AS in women and underscores the need for sex-specific approaches in managing AS and heart failure. Additionally, women have higher rates of hypertension, which may further influence their ventricular remodeling and AS progression [91].

Collectively, these sex-specific differences in presentation, valve pathology, remodeling, comorbidity burden, frailty, and treatment response highlight the need for tailored diagnostic and therapeutic strategies to improve outcomes in women with AS.

Emerging evidence from microbiota and metabolite research suggests potential contributors to these sex-specific patterns, although not directly established in AS. Moreover, sex-related differences in AS phenotypes may be partly associated with microbiota-related metabolic profiles. Women’s lower circulating TMAO and IS levels, partly driven by estrogen-modulated microbiota composition (higher *Akkermansia* and *Bifidobacterium*, lower *Firmicutes*), may be associated with less pronounced calcific remodeling, delaying the onset of overt hemodynamic obstruction. In contrast, in men, the difference in gut microbiota composition may be associated with higher TMAO/IS levels and a more rapid calcific phenotype, potentially contributing to earlier clinical presentation. despite similar baseline comorbidities. The influence of the main bacteria on the metabolites is summarized in the Table 1.

**Table 1:** Overview of the gut microbiota with their primary functions and the metabolites they influence in women and men

	**Gut microbiota**	**Primary function(s)**	**Metabolite(s) they influence**	Ref.
**More prevalent in women**	*Akkermansia muciniphila*	- crucial for healthy physiological functions - enhances integrity of gut barrier, regulates immune reaction, lessens inflammatory response - increases mucus secretion - reverses increased fat mass, adipose tissue inflammation, and insulin resistance, supports reproduction of butyrate-producing bacteria	- acceleration of intestinal epithelial cells development due to interactions with SCFAs and bile acids, peptidoglycan and lipopolysaccharide - impact on genetic expression of ZO-1, Occludin, and Claudin3 - suppresses production of inflammatory molecules such as TNF-α and IL-8 - increases number of goblet cells - upregulation of expression of NLRP6 inflammasome - acceleration of autophagy in goblet cells - stimulation of growth of mucin-secreting goblet cells	^[93]^
	*Bifidobacterium*	- improvement of intestinal epithelial homeostasis and decrease of intestinal permeability - reduces inflammation, increases more fibrotic remodeling process	- SCFAs - production of Lactate and Acetate, B-vitamin (B9), Indole lactic acid - production of GABA - reduction of TMAO production	^[93, 94, 95]^
	*Ruminococcus*	- degradation and fermentation of complex carbohydrates	- production of more SCFAs and less TMAO and IS - polysaccharides like cellulose - carbohydrate active enzymes	^[125]^
	members of *Lactobacillales*	- enhancing intestinal barrier defence by promoting mucus secretion	- upregulation of mucosal protective *MUC2* gene in colonic epithelial cells - SCFAs - production of hydrogen peroxide and lactic acid - bacteriocins - increase of sIgA levels in the small intestine	^[126]^
	members of *Streptococcaceae*	- modulation of immune system	- production of SCFAs through dietary fiber fermentation - production of B vitamins - synthesis of Phosphotidylcholine through a rare host metabolite uptake pathway	^[124,134]^
	*Slackia* (*Actinobacteria*)	- influences host lipid and xenobiotic metabolism	- transformation of isoflavones (e.g. equol) - generation of dihydroresveratrol	^[135, 136, 137]^
	*Butyricimonas* (*Bacteroidetes*)	- negatively related to obesity - degradation of high molecular weight organic matter, fermentation of otherwise indigestible polysaccharides	- SCFAs like butyrate - indigestible polysaccharides - proteins and carbohydrates	^[138,139]^
	*Bacteroidetes phylum*	- degraders of polymeric organic matter	- polysaccharides	^[138,139]^
**More prevalent in men**	*Prevotella*	- fermentation of dietary fiber	- SCFAs, high production of propionate, lower production of butyrate - release of ester modifications from carbohydrates to facilitate degradation	^[140,141]^
	*Bacteroides*	- provides protection from pathogens - degradation of various kinds of glycans including diet derived and host glycans, polysaccharides - optimizes colonization and maintenance in the gut	- plant polysaccharides - capsular polysaccharide synthesis	^[142]^
	*Veillonella*	- lactate fermentation	- production of SCFAs (especially acetate and propionate) by lactate fermentation	^[143]^
	*Methanobrevibacter*	- detoxifying hydrogen from bacterial fermentations, converting into mechanically expelled gaseous methane (CH_4_)	- hydrogen - production of metabolic byproduct while producing ATP via methanogenesis	^[144,145]^
	*Coprococcus catus*	- exerts anti-inflammatory effects on specific immune cells of the brain, and influences our reward centers in the brain - fiber fermentation - improvement of epithelial barrier function	- production of SCFAs acetate and butyrate (has multiple butyrate production pathways)	^[146,147]^
	*Acinetobacter* (*Proteobacteria*)	- fermentation process	- vitamins - peptides - polysaccharides	^[148]^
	*Dorea* (*Firmicutes*)	- fermentation of carbohydrates - reduction of inflammation	- production of SCFA butyrate, acetate and lactate	^[149]^
	*Ruminococcus* (*Firmicutes*)	see above	see above	
	*Megamonas* (*Firmicutes*)	- Gut microbial myo-inositol degradation - inhibits fatty acid transport - promotes intestinal lipid absorption - fermentation	- production of propionic acid - activation of the *AMPK* pathways by major fermentation end product propionic acid	^[150,151]^
	*Paraprevotella* (*Bacteroidetes*)	- maintaining intestinal homeostasis by promoting trypsin autolysis - protects host epithelium - protects against infection - fermentation and degradation	- production of secondary bile acid - production of acetate, glucose, glutamate, D-mannose, L-glutamate, maltose	^[152,153]^

Taken together, these observations support a hypothesis-generating framework linking gut microbiota–derived metabolites with sex-specific clinical expression of aortic stenosis. Variations in circulating metabolites such as TMAO and IS, which are influenced by diet, hormonal milieu, and microbiota composition [73, 75], may be associated not only with valvular changes but also with differences in ventricular remodeling and clinical outcomes. For instance, higher TMAO levels have been associated with the presence of severe AS and with increased mortality following transcatheter aortic valve replacement, suggesting a potential link between microbiota-derived metabolites and disease severity and prognosis [92]. In this context, sex-related differences in metabolite exposure could contribute to the distinct patterns of left ventricular adaptation observed between men and women, including the predominance of concentric remodeling and low-flow states in women versus more eccentric remodeling in men. Such differences may also be associated with symptom burden, timing of clinical presentation, and outcomes after intervention.

Rather than reflecting a direct causal pathway, these interactions likely represent a multifactorial and dynamic process in which microbiota composition, metabolite levels, and host-specific factors converge to shape disease expression. This framework may help generate testable hypotheses to explain why women more frequently develop fibrotic, low-flow phenotypes with preserved ejection fraction, whereas men tend to present earlier with more calcific disease and different remodeling patterns. However, direct evidence linking gut microbiota–derived metabolites to sex-specific clinical presentation and outcomes in AS remains limited and warrants further investigation in dedicated translational and clinical studies.In addition to the established sex-related differences in clinical presentation, an emerging body of literature suggests that the gut microbiota may represent an upstream modifier of these phenotypes through its effects on metabolite production, intestinal barrier integrity, systemic inflammation, and host endocrine signaling. Sex-related differences in gut microbial composition have been reported across healthy and disease cohorts, and are influenced by age, diet, and hormonal status [41, 49, 53, 54]. This is relevant in AS because microbiota-derived metabolites such as TMAO and IS have been implicated in pathways linked to valvular inflammation, fibrosis, and calcification [43, 58, 59, 69, 70]. Moreover, one of the few AS-related microbiota studies reported that the intestinal microbial profile associated with cardiac valve calcification differs from that observed in coronary artery disease, with taxa such as *Prevotella* being highlighted in this context [65]. Conversely, taxa including *Akkermansia muciniphila* and *Bifidobacterium* are associated with intestinal barrier support and beneficial metabolic functions [93–95]. Since dietary patterns also differ by sex at the population level, with women generally consuming more fruit- and vegetable-rich diets than men [55, 73, 74], sex-specific microbiota configurations may partly shape the circulating metabolite milieu relevant to AS. Although direct evidence linking gut microbiota composition to sex-specific clinical presentation or outcomes in AS remains limited, these observations support a hypothesis-generating framework in which microbial ecology may contribute to differences in disease expression, timing of presentation, and remodeling patterns between women and men [43, 77, 78].».

## Potential impact of sex and microbiota differences on the pathophysiology of AS

### Differences in VEC and VIC biology

VECs form a protective monolayer on both the ventricular and fibrosa surfaces of the aortic valve, playing essential roles in nitric oxide production, immune modulation, and VIC replenishment (Fig. 1). Their behavior is influenced by mechanical forces—shear stress on the ventricularis and oscillatory shear on the fibrosa—making them vulnerable to endothelial–mesenchymal transition (EndMT) and pro-osteogenic differentiation under pathological conditions. Notably, sex differences in VEC biology remain largely unexplored, though a recent study on porcine AVs revealed that male VECs exhibit higher proliferative activity than female VECs in vitro, possibly due to elevated thrombospondin 2 secretion by female VECs [96]. This reduced proliferative capacity in females may limit VEC renewal and repair, potentially contributing to the enhanced fibrotic remodeling observed in women with AS. In contrast, greater VEC proliferation in males could facilitate ongoing inflammation or maladaptive repair, linking endothelial dynamics to sex-specific disease trajectories [97].

Male porcine VICs cultured in osteogenic conditions form larger and denser calcium nodules than female VICs, and male rat VICs show higher expression of early osteogenic markers. These findings indicate that male VICs are more likely to undergo calcification, whereas female VICs may have a greater tendency for fibrotic events, such as increased metabolic activity and collagen production [98]. Additionally, female VICs exhibit higher levels of vascular endothelial growth factor A (VEGF-A) and basic fibroblast growth factor (FGF-2) secretion, which may influence angiogenesis in a sex-specific manner [99]. In addition the fibrotic predominance in women aligns with microbiota profiles enriched for anti-inflammatory taxa and lower production of pro-calcific metabolites such as TMAO and IS. These lower levels may preferentially activate pro-fibrotic TGF-β signaling over bone morphogenetic protein (BMP)-driven calcification. Conversely, the higher TMAO/IS burden seen in men, due to *Firmicutes*- and *Prevotella*-rich gut microbiota, likely synergizes with their more osteogenic VIC phenotype to accelerate calcific deposition. However, observations still show discrepancies in the role of VICs in sex-specific calcification and fibrosis, and further research is needed to clarify the results [100].

### Differences in signaling pathway regulation

Sex-specific immune and metabolic pathways contribute to distinct valvular remodeling phenotypes in AS. In stenotic aortic valves, women exhibit greater mesenchymal cell signatures, while men display enhanced infiltration of immune cells, including macrophages, monocytes, T cells, and B cells. This cellular divergence is further reflected at the molecular level: in vitro, VICs from females counteract osteogenic stimuli such as interferon-α and lipopolysaccharide via the Akt signaling pathway, suggesting a sex-specific regulation of inflammatory and osteogenic responses [101, 102]. In parallel, Prabutzki et al. [103] have uncovered a marked sexual dimorphism in aortic valve lipid composition during fibro-calcific disease progression. Female valves are enriched in sphingomyelins and ceramides, sphingolipids associated with fibrotic remodeling, possibly through the activation of TGF-β signaling, which is more active in women with AS [104]. In contrast, male valves accumulate more polyunsaturated triglycerides and show a greater loss of cardiolipins and phosphatidylserines, lipids whose depletion reflects mitochondrial damage, oxidative stress, elements that are described to promote calcification [103]. Recent studies using hydrogel biomaterials mimicking valve stiffness have also shown that female VICs exhibit higher basal and stiffness-induced activation of alpha-smooth muscle actin (α-SMA), a hallmark of myofibroblast transition, than male VICs. This enhanced activation in females is partly driven by Rho-associated protein kinase (ROCK) signaling and influenced by genes escaping X-chromosome inactivation, such as Bone Marrow Tyrosine Kinase Gene in Chromosome X and Steroid Sulfatase (Microsomal), Isozyme S, suggesting intrinsic sex-linked regulatory mechanisms of fibrosis [87]. Microbiota-derived metabolites may influence these intrinsic VIC/VEC behaviors. TMAO, more abundant in men, can enhance osteogenic gene expression in VICs via PI3K/Akt and STAT1-ERK-HIF1α pathways, amplifying calcification potential. In contrast, the metabolite profile typical of women, with reduced TMAO and increased SCFA-producing bacteria, may support myofibroblast activation and collagen synthesis, potentially contributing to a more fibrotic valvular remodeling pattern.

Further, in the RAAS (Renin–Angiotensin–Aldosterone System), aldosterone promotes calcification in male VICs while driving fibrosis in female VICs. Angiotensin II, acting primarily via Angiotensin II type 1 (AT1) receptors in the aortic valve, triggers inflammation, fibrosis, and TGF-β expression, though the sex-specific nuances of these effects remain unclear [105]. Similarly, Notch signaling plays a protective role by suppressing osteogenic markers such as BMP2 and RUNX2 [106], thereby limiting calcification in female VICs. Downregulation of Notch1 is associated with calcified AS, and while sex-specific differences are not yet fully established, variations in Notch activity may contribute to distinct disease phenotypes like sex-specific modulation of the Notch pathway in fibroblasts in response to hyperoxia [107, 108]. Another player, the TGF-β/BMP signaling axis [109] further illustrates this balance: TGF-β can be pro-fibrotic or anti-calcific depending on the cellular context. In females, its upregulation tends to promote fibrosis, whereas BMPs—members of the same superfamily—are strongly pro-calcific and show greater activity in males. The interplay between TGF-β and BMP signaling likely shapes the sex-dependent patterns of fibrosis and calcification observed clinically [109]. Finally, the Wnt/β-Catenin pathway [110, 111] is notably active in severe AS, driving both fibrosis and calcification. Several Wnt-related genes are upregulated in female aortic valves, potentially under estrogenic regulation, while testosterone also influences this pathway, underscoring a broader hormonal modulation of disease expression.

In addition, gut-derived metabolites may intersect with these pathways: TMAO can amplify RAAS-induced oxidative stress, IS may potentiate Receptor Activator of Nuclear Factor κB Ligand (RANKL)-mediated osteogenesis, and SCFAs from beneficial taxa can modulate Wnt/β-catenin signaling toward fibrosis rather than calcification. Thus, the hormonal modulation of microbiota composition may indirectly steer pathway dominance in a sex-specific manner.

In summary, RAAS, TGF-β, and Wnt/β-catenin are primarily associated with fibrosis, especially in females, whereas RANK/RANKL/OPG, BMP signaling, and Notch1 downregulation contribute more directly to calcification, predominantly observed in males. Together, these observations suggest that gut microbiota–derived metabolites may represent an additional layer of regulation interacting with hormonal and intrinsic cellular pathways, potentially contributing to the sex-dependent balance between fibrotic and calcific remodeling in aortic stenosis, although this integrative model remains hypothetical and requires further investigation.

### Specific impact of estrogens and testosterone

Sex hormones appear to play a central role in the sex-specific pathophysiology of AS and might contribute to the difference in the composition and remodeling of ECM. Estrogens, especially 17β-estradiol, have been shown to exert cardioprotective effects by modulating inflammation, vascular remodeling, and ECM turnover, particularly through inhibition of fibroblast proliferation and Matrix Metalloproteinase-2 (MMP2) expression in females [112, 113], along with a lower activity level of collagenase and gelatinase, contributing to the accumulation of ECM [114]. Furthermore, 17β-estradiol inhibits the transcription of MMP2 via the MAPK-ERK1/2 signaling pathway in cardiac fibroblasts, indicating a potential role of estrogens in modulating ECM remodeling in a sex-specific manner [115]. These protective effects seem to be sex-specific, as β-estradiol reduces VIC proliferation and fibrosis in females but not in males [98]. Estrogens also generally downregulate RAAS activity and exert cardioprotective effects before menopause, whereas testosterone amplifies RAAS-driven vasoconstriction and fibrosis through collagen accumulation [116]. This hormonal influence extends into the RANK/RANKL/OPG pathway [117], a central driver of calcification that is more active in males, where elevated RANKL expression correlates with greater aortic valve calcification. Estrogens can inhibit RANKL activity [118], potentially explaining the reduced calcification typically observed in premenopausal females. This protection is further amplified by the ‘estrobolome’, where estrogen-primed bacteria (like *Akkermansia*) maintain systemic estradiol levels via β-glucuronidase activity, while simultaneously reducing the translocation of pro-inflammatory LPS, a known trigger for valvular TLR4 signaling [119]. Conversely, testosterone is increasingly recognized as a pro-calcific factor, associated with higher expression of osteogenic genes and worsening of AS hemodynamics in male models, possibly through activation of the androgen receptor, which is overexpressed in stenotic male valves [120–122] andsynergy with gut-derived metabolites such as IS and TMAO. These metabolites, enriched in the male-associated microbiota, activate the Aryl hydrocarbon Receptor and NFkB pathways, that could directly upregulating osteogenic genes like Runx2 in male VICs [58, 123]. The levels of glycosaminoglycans, collagen I, and activated MMP2 are higher compared to female individuals. Although the exact mechanisms remain to be fully elucidated, the differential expression and action of sex hormone receptors in the aortic valve suggest a direct influence on disease development and progression [121].

Taken together, sex hormones, the gut microbiota, and microbiota-derived metabolites likely form an interconnected regulatory axis influencing extracellular matrix remodeling in AS. Rather than acting as independent drivers, these systems may converge on shared signaling pathways governing fibrosis and calcification, with sex hormones shaping both microbial composition and host tissue responsiveness to circulating metabolites. This integrated framework provides a plausible biological context for the observed sex-dependent differences in valvular remodeling, although direct mechanistic evidence linking these layers in AS remains limited and should be further explored in dedicated experimental and translational studies.Beyond intrinsic sex differences in valve biology and hormonal regulation, the gut microbiota may provide an additional upstream layer influencing the pathophysiology of AS. The microbiota affects the host through several interconnected mechanisms, including production of bioactive metabolites, modulation of epithelial permeability, regulation of immune homeostasis, and crosstalk with sex hormone metabolism [34, 41, 43, 49, 51, 124]. This may be particularly relevant in AS, where the major downstream processes driving disease progression-endothelial dysfunction, inflammatory cell recruitment, VIC activation, fibrosis, and calcification-are all susceptible to modulation by microbiota-derived metabolites such as TMAO and IS [58, 59, 64, 69, 70]. Experimental data already support direct effects of these metabolites on valvular cells: TMAO promotes osteogenic differentiation and fibrosis in aortic valve models [58, 59], whereas IS induces endothelial injury, endothelial-to-mesenchymal transition, calcification, and inflammatory activation [69, 70]. In parallel, sex-dependent microbial features described in broader microbiome studies, together with hormone–microbiota interactions mediated through the estrobolome, suggest that women and men may be exposed to distinct microbial-metabolic environments over the course of disease [41, 49, 51, 53]. Taxa such as *Prevotella*, highlighted in AS-associated microbiota studies [65], as well as *Akkermansia*, *Bifidobacterium*, and Lactobacillales, which are linked to barrier integrity and immune modulation [93–95, 125, 126], further support the biological plausibility of this concept. At present, however, these interactions remain insufficiently validated in sex-stratified AS cohorts, and should therefore be interpreted as a plausible but still hypothesis-generating framework rather than an established causal model.

### Lessons from animal models

In murine models of AS, male mice showed greater disease progression and increased expression of pro-calcific genes (e.g., RunX2), while female mice displayed downregulation of such genes (e.g., ALPL), despite comparable hemodynamic progression. Castration reduced AS progression in males, while testosterone reintroduction partially restored it. In females, neither ovariectomy nor estrogen supplementation significantly altered disease progression [127]. Mice after ovariectomy showed a significantly increased left ventricle mass without fibrosis. The aortic valve peak velocity was slightly increased, but there was no difference in mean pressure gradient across the aortic valve, compared with control animals [128]. Another very recent study also shows that the occurrence or progression of AVS does not differ significantly between mice after ovariectomy and control animals [129].

Moreover, sex-specific mitochondrial function differences were identified in both humans with AS and murine models. Female hearts showed higher mitochondrial respiration, which decreased after gonadectomy. Treatment with 17β-estradiol or an ERβ agonist restored mitochondrial function in female mice, but not in males, highlighting the specific role of estrogen and ERβ in cardiac energetics and potentially AS pathophysiology [130]. Together, testosterone might promote calcification and disease progression, while estrogens, particularly 17β-estradiol, might be protective by reducing fibrosis, inhibiting VIC proliferation, and preserving mitochondrial function. These opposing effects may contribute to the distinct patterns of calcific remodeling in men and more fibrotic valvular remodeling in women.

Together, current evidence shows that sex differences in aortic valve pathophysiology arise from multiple, interconnected layers. At the cellular level, male VICs and VECs tend to display greater osteogenic potential and proliferative activity, favoring calcification, while female cells more often engage pro-fibrotic programs. These intrinsic tendencies are reinforced by sex-specific regulation of signaling pathways: women show stronger TGF-β, RAAS, and Wnt/β-catenin–driven fibrotic responses, whereas men exhibit enhanced RANK/RANKL/OPG and BMP signaling that accelerates calcification. Sex hormones provide an additional regulatory axis—estrogens dampening inflammation, ECM degradation, and RANKL-mediated calcification, testosterone amplifying RAAS and osteogenic signaling—while also shaping gut microbiota composition and metabolite profiles (e.g., TMAO, IS, SCFAs) that further bias valve remodeling. Finally, findings from animal models and human tissue studies underline the above described findings: calcification predominates in males, fibrosis in females. Within this framework, sex-specific differences in gut microbiota may act as an upstream modulator integrating hormonal influences with circulating metabolite profiles, thereby potentially contributing to the observed bias toward pro-calcific versus pro-fibrotic signaling environments in the aortic valve. This remains a hypothesis that warrants further mechanistic investigation.

### Integrating gut microbiota, sex difference and signaling pathways in AS

Taken together, these observations support a hypothesis-generating framework in which gut microbiota–derived metabolites may contribute to sex-specific modulation of the signaling pathways involved in aortic stenosis. Sex-related differences in gut microbiota composition and function may lead to distinct circulating profiles of metabolites such as TMAO, IS, and SCFAs, which have been shown in experimental models to interact with key pathways implicated in valvular remodeling, including RAAS, TGF-β, Wnt/β-catenin, and RANK/RANKL/OPG signaling [58, 131, 132]. If we extrapolate the data from coronary artery disease or CKD, in women, a metabolite milieu relatively enriched in SCFAs and lower in TMAO which promotes pro-oxidative and pro-calcific environment may preferentially align with fibrotic signaling programs, whereas in men higher exposure to TMAO and IS may be more consistent with activation of osteogenic and inflammatory pathways via Wnt/β-catenin and NF-κB signaling [123]. Importantly, these metabolites are unlikely to act as primary disease drivers, but rather as modulators of sex-biased cellular responses within an already heterogeneous valvular environment. In this context, gut microbiota–host interactions may help amplify or attenuate pre-existing sex differences in signaling pathway activity, thereby contributing to the divergence between fibrotic and calcific phenotypes. This integrative framework remains speculative and hypothesis-driven, as direct mechanistic evidence linking sex-specific microbiota–metabolite profiles to differential valvular signaling in aortic stenosis is currently limited and warrants further experimental validation (Table 2).

**Table 2:** Level of evidence for mechanistic links between TMAO, IS and sex-specific phenotypes in aortic stenosis

Proposed Mechanistic Link	Evidence in AS Patients	Evidence in other Human CVD/CKD	Experimental (In vitro / Animal)	Level of Evidence	Key References
**TMAO-Related Pathways**					
**Valvular Calcification** (PI3K/Akt, STAT1 pathways)	**Absent** (No direct human cohort data)	**Established** (Association with remodeling)	**Strong** (VIC/VEC osteogenic differentiation)	**Moderate**	^[58, 59, 60, 61, 62, 63]^
**Macrophage Activation** (NF-κB, M1 polarization)	**Inferred** (Based on infiltration in male valves)	**Established** (Pro-inflammatory effects)	**Strong** (In vitro mechanistic studies)	**Moderate**	^[64,^ ^59]^
**Sex-Specific Levels** (Lower in women)	**Absent** (Not studied in AS populations)	**Established** (General & CAD populations)	**N/A**	**Inferred**	^[60,61]^, ^[65, 67, 68]^
**Indoxyl Sulfate (IS) Pathways**					
**Endothelial & Valvular Injury** (NKD2, IL-6)	**Limited** (Translational studies)	**Established** (CKD & Atherosclerosis)	**Strong** (In vitro + In vivo)	**Moderate**	^[69, 70, 71]^
**Sex-Specific Levels** (Higher in men)	**Absent** (Explicitly missing in AS)	**Confirmed** (CAD & CKD cohorts)	**N/A**	**Inferred**	^[72]^
**Integrated Gut-Heart Axis**					
**Sex x Microbiota x AS Phenotype**	**Absent** (Direct link not established)	**Absent**	**Absent** (Hypothesis-generating)	**Speculative**	

AS: Aortic stenosis; VIC: Valvular interstitial cell; VEC: Valvular endothelial cell CVD: Cardiovascular disease; CKD: Chronic kidney disease; WD: Western diet; MD: Mediterranean diet

### Limitations

Several limitations should be acknowledged when interpreting the current evidence linking sex differences, gut microbiota, and AS. First, sex-stratified microbiome and metabolomic data specifically in AS remain extremely scarce, with most available evidence derived from studies in cardiovascular, metabolic, or renal diseases rather than valve-specific cohorts. This limits direct inference to valvular pathology and necessitates cautious extrapolation.

Second, findings across studies are likely influenced by multiple confounding factors, including diet, medication use (particularly statins, antibiotics, and proton pump inhibitors), comorbidities such as chronic kidney disease and diabetes, and age-related microbiome shifts. These variables are not consistently controlled across studies and may significantly affect both microbial composition and metabolite levels.

Third, substantial methodological heterogeneity exists across microbiome studies, including differences in sequencing platforms, bioinformatic pipelines, sample types, and metabolomic techniques. This variability limits direct comparability and may contribute to inconsistencies in reported taxa and metabolite associations.

Fourth, the majority of available data are cross-sectional, precluding causal inference and limiting the ability to determine whether microbiota alterations are a driver, consequence, or bystander of disease progression. Longitudinal studies in AS are particularly lacking.

Finally, there is a clear absence of direct mechanistic validation in human valvular tissue linking microbiota-derived metabolites to sex-specific cellular responses in VICs or VECs. Most proposed mechanisms remain inferred from in vitro or animal models in other disease contexts, highlighting an important translational gap.

Together, these limitations underscore the exploratory and hypothesis-generating nature of the current framework and emphasize the need for well-designed, sex-stratified, longitudinal, and mechanistic studies in AS.

## Conclusion and outlook

The review explores the complex interplay between sex differences, gut microbiota, and the pathophysiology of AS. It highlights that men tend to experience a more severe form of AS, which may be linked to sex-specific differences in gut microbiota composition. Men often have higher levels of *Prevotella*, a bacterial species associated with the production of TMAO, a metabolite linked to valve fibrosis and calcification. In contrast, women typically exhibit more protective bacteria and lower TMAO production, which might offer a protective effect against AS progression (Fig. 2).

While causality remains to be established, converging evidence supports a model in which sex hormones influence gut microbiota composition and metabolite production, which in turn could modulate sex-specific differences in VICs, VECs, immune cell infiltration, and signaling pathways. Importantly, these insights open the door to novel, sex-tailored interventions for AS prevention and progression control. Clinically, gut microbiota modulation could be pursued through dietary strategies (e.g., MD patterns, reduced intake of TMAO precursors such as red meat and egg yolks), targeted pre- and probiotics, or even microbiota-directed pharmacological agents. Given that AS is often diagnosed years before symptoms require intervention, the preclinical or early clinical stage represents a realistic time window for implementing such strategies.

Regional dietary patterns may further shape these opportunities: for instance, the traditional Japanese diet, low in red meat and rich in fermented foods, has been associated with distinct gut microbiota profiles and lower cardiovascular calcification rates, whereas certain South American diets high in red meat could, in theory, favor a more TMAO-promoting microbiome. Integrating microbiome analysis into AS risk stratification could help identify high-risk patients, enabling early lifestyle or microbiota-targeted interventions before irreversible valvular damage occurs especially in high income countries where degenerative AS is the most prevalent while rheumatic AS is more encountered in low income countries [133]. The prevalence of this condition increases sharply with age and is highest in Australasia, Europe, and North America [133]. Its geographical distribution parallels that of major atherosclerotic risk factors, such as smoking, hypertension, hypercholesterolemia, and elevated BMI which are strongly associated with calcific aortic stenosis.

Future studies are needed to directly test the proposed interactions between sex, gut microbiota-derived metabolites, and aortic valve remodeling. At the cellular level, sex-stratified in vitro experiments using male- and female-derived valvular interstitial and endothelial cells would be particularly informative, allowing direct comparison of their transcriptional and phenotypic responses to key metabolites such as TMAO, IS, and SCFAs. Such models could help determine whether sex-specific differences exist in osteogenic versus fibrotic activation thresholds, as well as in the engagement of signaling pathways such as PI3K/Akt, TGF-β, and RANKL. At the preclinical level, existing animal models of elevated gut-derived metabolites, such as chronic IS exposure in kidney disease models, could be adapted to investigate valvular remodeling in both male and female animals, enabling assessment of sex-dependent susceptibility to calcification and fibrosis under controlled metabolic conditions. Germ-free or antibiotic-treated mouse models colonized with defined microbial communities enriched in TMA-producing taxa could further allow mechanistic dissection of microbiota-driven effects on valve biology in a sex-specific context. Finally, integrating these experimental approaches with longitudinal human cohorts measuring microbiome composition, circulating metabolites, and serial imaging of valve calcification would provide the translational bridge required to validate causality and refine microbiome-informed risk stratification strategies in aortic stenosis.

## Data Availability

No datasets were generated or analysed during the current study.

## Abbreviations

Α-SMA: Alpha-smooth muscle actin
AS: Aortic stenosis
AT1: Angiotensin II type 1 receptor
ATP: Adenosine Triphosphate
AV: Aortic valve
AVR: Aortic valve replacement
BMP: Bone morphogenetic protein
CAS: Calcific aortic stenosis
CT: Computed tomography
CKD: Chronic kidney disease
ECM: Extracellular matrix
EndMT: Endothelial–mesenchymal transition
ER: Endoplasmic reticulum
FGF-2: Fbroblast growth factor
IL-6: Interleukin-6
IS: Indoxyl sulfate
LV: Left ventricular
MD: Mediterranean diet
MMP2: Matrix Metalloproteinase-2
NLRP6: NOD-like receptor family pyrin domain containing 6
RAAS: Renin–Angiotensin–Aldosterone System
SAVR: Surgical aortic valve replacement
SCFA: Short-chain fatty acids
TAVI: Transcatheter aortic valve implantation
TMA: Trimethylamine-N, precursor of TMAO
TMAO: Trimethylamine-N-oxide
RANKL: Receptor Activator of Nuclear Factor κB Ligand
ROCK: Rho-associated protein kinase
USA: United States of America
VECs: Valve endothelial cells
VEGF-A: Vascular endothelial growth factor A
VICs: Valve interstitial cells
WD: Western diet
ZO-1: Zonula Occludens-1

## References

[1] Vahanian A, Beyersdorf F, Praz F, Milojevic M, Baldus S, Bauersachs J, Capodanno D, Conradi L, De Bonis M, De Paulis R, Delgado V, Freemantle N, Gilard M, Haugaa KH, Jeppsson A, Jüni P, Pierard L, Prendergast BD, Sádaba JR, ESC National Cardiac Societies. 2021 ESC/EACTS Guidelines for the management of valvular heart disease: Developed by the Task Force for the management of valvular heart disease of the European Society of Cardiology (ESC) and the European Association for Cardio-Thoracic Surgery (EACTS). Eur Heart J. 2022;43(7):561–632. 10.1093/eurheartj/ehab395. 34453165 10.1093/eurheartj/ehab395

[2] Carabello BA. Clinical practice. Aortic stenosis. N Engl J Med. 2002;346(9):677–82. 10.1056/NEJMcp010846. 11870246 10.1056/NEJMcp010846

[3] Nkomo VT, Gardin JM, Skelton TN, Gottdiener JS, Scott CG, Enriquez-Sarano M. Burden of valvular heart diseases: A population-based study. Lancet (London England). 2006;368(9540):1005–11. 10.1016/S0140-6736(06)69208-8. 16980116 10.1016/S0140-6736(06)69208-8

[4] Iung B, Baron G, Butchart EG, Delahaye F, Gohlke-Bärwolf C, Levang OW, Tornos P, Vanoverschelde J-L, Vermeer F, Boersma E, Ravaud P, Vahanian A. A prospective survey of patients with valvular heart disease in Europe: The Euro Heart Survey on Valvular Heart Disease. Eur Heart J. 2003;24(13):1231–43. 10.1016/s0195-668x(03)00201-x. 12831818 10.1016/s0195-668x(03)00201-x

[5] Passik CS, Ackermann DM, Pluth JR, Edwards WD. (1987). Temporal changes in the causes of aortic stenosis: A surgical pathologic study of 646 cases. *Mayo Clinic Proceedings*, *62*(2), 119–123. 10.1016/s0025-6196(12)61880-1 10.1016/s0025-6196(12)61880-1 3807436

[6] Collins MJ, Butany J, Borger MA, Strauss BH, David TE. Implications of a congenitally abnormal valve: A study of 1025 consecutively excised aortic valves. J Clin Pathol. 2008;61(4):530–6. 10.1136/jcp.2007.051904. 17965218 10.1136/jcp.2007.051904

[7] American College of Cardiology/American Heart Association Task Force on Practice Guidelines, Society of Cardiovascular Anesthesiologists, Society for Cardiovascular Angiography and Interventions, Society of Thoracic Surgeons, Bonow RO, Carabello BA, Kanu C, de Leon AC, Faxon DP, Freed MD, Gaasch WH, Lytle BW, Nishimura RA, O’Gara PT, O’Rourke RA, Otto CM, Shah PM, Shanewise JS, Smith SC, Riegel B. ACC/AHA 2006 guidelines for the management of patients with valvular heart disease: A report of the American College of Cardiology/American Heart Association Task Force on Practice Guidelines (writing committee to revise the 1998 Guidelines for the Management of Patients With Valvular Heart Disease): developed in collaboration with the Society of Cardiovascular Anesthesiologists: endorsed by the Society for Cardiovascular Angiography and Interventions and the Society of Thoracic Surgeons. Circulation. 2006;114(5):e84–231. 10.1161/CIRCULATIONAHA.106.176857. 16880336 10.1161/CIRCULATIONAHA.106.176857

[8] Freeman RV, Otto CM. Spectrum of calcific aortic valve disease: Pathogenesis, disease progression, and treatment strategies. Circulation. 2005;111(24):3316–26. 10.1161/CIRCULATIONAHA.104.486738. 15967862 10.1161/CIRCULATIONAHA.104.486738

[9] Stewart BF, Siscovick D, Lind BK, Gardin JM, Gottdiener JS, Smith VE, Kitzman DW, Otto CM. Clinical factors associated with calcific aortic valve disease. Cardiovascular Health Study. J Am Coll Cardiol. 1997;29(3):630–4. 10.1016/s0735-1097(96)00563-3. 9060903 10.1016/s0735-1097(96)00563-3

[10] Otto CM, Kuusisto J, Reichenbach DD, Gown AM, O’Brien KD. Characterization of the early lesion of „degenerative valvular aortic stenosis. Histological and immunohistochemical studies. Circulation. 1994;90(2):844–53. 10.1161/01.cir.90.2.844. 7519131 10.1161/01.cir.90.2.844

[11] Yetkin E, Waltenberger J. Molecular and cellular mechanisms of aortic stenosis. Int J Cardiol. 2009;135(1):4–13. 10.1016/j.ijcard.2009.03.108. 19386374 10.1016/j.ijcard.2009.03.108

[12] Carabello BA, Paulus WJ. Aortic stenosis. Lancet (London England). 2009;373(9667):956–66. 10.1016/S0140-6736(09)60211-7. 19232707 10.1016/S0140-6736(09)60211-7

[13] Leopold JA. Cellular mechanisms of aortic valve calcification. Circ Cardiovasc Interv. 2012;5(4):605–14. 10.1161/CIRCINTERVENTIONS.112.971028. 22896576 10.1161/CIRCINTERVENTIONS.112.971028 PMC3427002

[14] Mohler ER, Sheridan MJ, Nichols R, Harvey WP, Waller BF. Development and progression of aortic valve stenosis: Atherosclerosis risk factors–a causal relationship? A clinical morphologic study. Clin Cardiol. 1991;14(12):995–9. 10.1002/clc.4960141210. 1841025 10.1002/clc.4960141210

[15] Clavel M-A, Messika-Zeitoun D, Pibarot P, Aggarwal SR, Malouf J, Araoz PA, Michelena HI, Cueff C, Larose E, Capoulade R, Vahanian A, Enriquez-Sarano M. The complex nature of discordant severe calcified aortic valve disease grading: New insights from combined Doppler echocardiographic and computed tomographic study. J Am Coll Cardiol. 2013;62(24):2329–38. 10.1016/j.jacc.2013.08.1621. 24076528 10.1016/j.jacc.2013.08.1621

[16] Aggarwal SR, Clavel M-A, Messika-Zeitoun D, Cueff C, Malouf J, Araoz PA, Mankad R, Michelena H, Vahanian A, Enriquez-Sarano M. Sex differences in aortic valve calcification measured by multidetector computed tomography in aortic stenosis. Circ Cardiovasc Imaging. 2013;6(1):40–7. 10.1161/CIRCIMAGING.112.980052. 23233744 10.1161/CIRCIMAGING.112.980052

[17] Thaden JJ, Nkomo VT, Suri RM, Maleszewski JJ, Soderberg DJ, Clavel M-A, Pislaru SV, Malouf JF, Foley TA, Oh JK, Miller JD, Edwards WD, Enriquez-Sarano M. Sex-related differences in calcific aortic stenosis: Correlating clinical and echocardiographic characteristics and computed tomography aortic valve calcium score to excised aortic valve weight. Eur Heart J. 2016;37(8):693–9. 10.1093/eurheartj/ehv560. 26508159 10.1093/eurheartj/ehv560 PMC6370337

[18] Simard L, Côté N, Dagenais F, Mathieu P, Couture C, Trahan S, Bossé Y, Mohammadi S, Pagé S, Joubert P, Clavel M-A. Sex-Related Discordance Between Aortic Valve Calcification and Hemodynamic Severity of Aortic Stenosis: Is Valvular Fibrosis the Explanation? Circul Res. 2017;120(4):681–91. 10.1161/CIRCRESAHA.116.309306. 10.1161/CIRCRESAHA.116.309306 27879282

[19] Cartlidge TR, Bing R, Kwiecinski J, Guzzetti E, Pawade TA, Doris MK, Adamson PD, Massera D, Lembo M, Peeters FECM, Couture C, Berman DS, Dey D, Slomka P, Pibarot P, Newby DE, Clavel M-A, Dweck MR. Contrast-enhanced computed tomography assessment of aortic stenosis. Heart. 2021;107(23):1905–11. 10.1136/heartjnl-2020-318556. 33514522 10.1136/heartjnl-2020-318556 PMC8600609

[20] Singh GK, Delgado V. Multimodality Imaging to Explore Sex Differences in Aortic Stenosis. Eur Cardiol Rev. 2022;17:e26. 10.15420/ecr.2022.26. 10.15420/ecr.2022.26 PMC9947932 36845220

[21] Maznyczka A, Tomii D, Angellotti D, Baekke PS, Nakase M, Samim D, Lanz J, Reineke D, Stortecky S, Gräni C, Windecker S, Pilgrim T. Fibrotic vs Calcific Aortic Stenosis: Characteristics and Outcomes in Patients Undergoing Transcatheter Aortic Valve Replacement. JACC: Cardiovasc Interventions. 2024;17(24):2969–71. 10.1016/j.jcin.2024.09.041. 10.1016/j.jcin.2024.09.041 39570229

[22] Diederichsen A, Lindholt JS, Møller JE, Gerke O, Rasmussen LM, Dahl JS. Sex Differences in Factors Associated With Progression of Aortic Valve Calcification in the General Population. Circ Cardiovasc Imaging. 2022;15(1):e013165. 10.1161/CIRCIMAGING.121.013165. 34983195 10.1161/CIRCIMAGING.121.013165

[23] Tchetche, D., Pibarot, P., Bax, J. J., Bonaros, N., Windecker, S., Dumonteil, N.,Nietlispach, F., Messika-Zeitoun, D., Pocock, S. J., Berthoumieu, P., Swaans, M. J.,Timmers, L., Rudolph, T. K., Bleiziffer, S., Leroux, L., Modine, T., van der Kley,F., Auffret, V., Tomasi, J., … for the RHEIA Investigators. (2025). Transcatheter vs. surgical aortic valve replacement in women: The RHEIA trial. *European Heart Journal*, *46*(22), 2079–2088. 10.1093/eurheartj/ehaf133. 10.1093/eurheartj/ehaf133 40171878

[24] Chandrasekhar J, Dangas G, Yu J, Vemulapalli S, Suchindran S, Vora AN, Baber U, Mehran R, STS/ACC TVT Registry. Sex-Based Differences in Outcomes With Transcatheter Aortic Valve Therapy: TVT Registry From 2011 to 2014. J Am Coll Cardiol. 2016;68(25):2733–44. 10.1016/j.jacc.2016.10.041. 28007135 10.1016/j.jacc.2016.10.041

[25] O’Connor SA, Morice M-C, Gilard M, Leon MB, Webb JG, Dvir D, Rodés-Cabau J, Tamburino C, Capodanno D, D’Ascenzo F, Garot P, Chevalier B, Mikhail GW, Ludman PF. Revisiting Sex Equality With Transcatheter Aortic Valve Replacement Outcomes: A Collaborative, Patient-Level Meta-Analysis of 11,310 Patients. J Am Coll Cardiol. 2015;66(3):221–8. 10.1016/j.jacc.2015.05.024. 26184614 10.1016/j.jacc.2015.05.024

[26] Saad M, Nairooz R, Pothineni NVK, Almomani A, Kovelamudi S, Sardar P, Katz M, Abdel-Wahab M, Bangalore S, Kleiman NS, Block PC, Abbott JD. Long-Term Outcomes With Transcatheter Aortic Valve Replacement in Women Compared With Men: Evidence From a Meta-Analysis. JACC Cardiovasc Intervent. 2018;11(1):24–35. 10.1016/j.jcin.2017.08.015. 10.1016/j.jcin.2017.08.015 29055767

[27] Williams M, Kodali SK, Hahn RT, Humphries KH, Nkomo VT, Cohen DJ, Douglas PS, Mack M, McAndrew TC, Svensson L, Thourani VH, Tuzcu EM, Weissman NJ, Kirtane AJ, Leon MB. Sex-related differences in outcomes after transcatheter or surgical aortic valve replacement in patients with severe aortic stenosis: Insights from the PARTNER Trial (Placement of Aortic Transcatheter Valve). J Am Coll Cardiol. 2014;63(15):1522–8. 10.1016/j.jacc.2014.01.036. 24561149 10.1016/j.jacc.2014.01.036

[28] Chandrasekhar J, Dangas G, Yu J, Vemulapalli S, Suchindran S, Vora AN, Baber U, Mehran R, STS/ACC TVT Registry. Sex-Based Differences in Outcomes With Transcatheter Aortic Valve Therapy: TVT Registry From 2011 to 2014. J Am Coll Cardiol. 2016;68(25):2733–44. 10.1016/j.jacc.2016.10.041. 28007135 10.1016/j.jacc.2016.10.041

[29] Bière L, Launay M, Pinaud F, Hamel J-F, Eltchaninoff H, Iung B, Laskar M, Leguerrier A, Gilard M, Furber A. Influence of sex on mortality and perioperative outcomes in patients undergoing TAVR: Insights from the FRANCE 2 registry. J Am Coll Cardiol. 2015;65(7):755–7. 10.1016/j.jacc.2014.11.044. 25677438 10.1016/j.jacc.2014.11.044

[30] Zhao Z-G, Liao Y-B, Peng Y, Chai H, Liu W, Li Q, Ren X, Wang X-Q, Luo X-L, Zhang C, Lu L-H, Meng Q-T, Chen C, Chen M, Feng Y, Huang D-J. Sex-related differences in outcomes after transcatheter aortic valve implantation: A systematic review and meta-analysis. Circ Cardiovasc Interv. 2013;6(5):543–51. 10.1161/CIRCINTERVENTIONS.113.000529. 24065446 10.1161/CIRCINTERVENTIONS.113.000529

[31] Conrotto F, D’Ascenzo F, Presbitero P, Humphries KH, Webb JG, O’Connor SA, Morice M-C, Lefèvre T, Grasso C, Sbarra P, Taha S, Omedè P, Marra G, Salizzoni W, Moretti S, D’Amico C, Biondi-Zoccai M, Gaita G, F., Marra S. Effect of gender after transcatheter aortic valve implantation: A meta-analysis. Ann Thorac Surg. 2015;99(3):809–16. 10.1016/j.athoracsur.2014.09.089. 25633460 10.1016/j.athoracsur.2014.09.089

[32] Stangl V, Baldenhofer G, Laule M, Baumann G, Stangl K. Influence of sex on outcome following transcatheter aortic valve implantation (TAVI): Systematic review and meta-analysis. J Interv Cardiol. 2014;27(6):531–9. 10.1111/joic.12150. 25156031 10.1111/joic.12150

[33] Qin, J., Li, R., Raes, J., Arumugam, M., Burgdorf, K. S., Manichanh, C., Nielsen,T., Pons, N., Levenez, F., Yamada, T., Mende, D. R., Li, J., Xu, J., Li, S., Li, D.,Cao, J., Wang, B., Liang, H., Zheng, H., … Wang, J. (2010). A human gut microbial gene catalogue established by metagenomic sequencing. *Nature*, *464*(7285), 59–65. 10.1038/nature08821. 10.1038/nature08821 PMC3779803 20203603

[34] Sommer F, Bäckhed F. The gut microbiota—Masters of host development and physiology. Nat Rev Microbiol. 2013;11(4):227–38. 10.1038/nrmicro2974. 23435359 10.1038/nrmicro2974

[35] Shanahan F. The colonic microbiota in health and disease. Curr Opin Gastroenterol. 2013;29(1):49–54. 10.1097/MOG.0b013e32835a3493. 23041677 10.1097/MOG.0b013e32835a3493

[36] Fukui H, Xu X, Miwa H. Role of Gut Microbiota-Gut Hormone Axis in the Pathophysiology of Functional Gastrointestinal Disorders. J Neurogastroenterol Motil. 2018;24(3):367–86. 10.5056/jnm18071. 29969855 10.5056/jnm18071 PMC6034676

[37] Sender R, Fuchs S, Milo R. Are We Really Vastly Outnumbered? Revisiting the Ratio of Bacterial to Host Cells in Humans. Cell. 2016;164(3):337–40. 10.1016/j.cell.2016.01.013. 26824647 10.1016/j.cell.2016.01.013

[38] Sender R, Fuchs S, Milo R. Revised Estimates for the Number of Human and Bacteria Cells in the Body. PLoS Biol. 2016;14(8):e1002533. 10.1371/journal.pbio.1002533. 27541692 10.1371/journal.pbio.1002533 PMC4991899

[39] Bischoff SC. Microbiota and aging. Curr Opin Clin Nutr Metab Care. 2016;19(1):26–30. 10.1097/MCO.0000000000000242. 26560527 10.1097/MCO.0000000000000242

[40] Salles N. Basic mechanisms of the aging gastrointestinal tract. Dig Dis (Basel Switzerland). 2007;25(2):112–7. 10.1159/000099474. 10.1159/000099474 17468545

[41] Kim YS, Unno T, Kim B-Y, Park M-S. Sex Differences in Gut Microbiota. World J Men’s Health. 2020;38(1):48–60. 10.5534/wjmh.190009. 30929328 10.5534/wjmh.190009 PMC6920072

[42] Arumugam, M., Raes, J., Pelletier, E., Le Paslier, D., Yamada, T., Mende, D. R.,Fernandes, G. R., Tap, J., Bruls, T., Batto, J.-M., Bertalan, M., Borruel, N., Casellas,F., Fernandez, L., Gautier, L., Hansen, T., Hattori, M., Hayashi, T., Kleerebezem,M., … Bork, P. (2011). Enterotypes of the human gut microbiome. *Nature*, *473*(7346), 174–180. 10.1038/nature09944. 10.1038/nature09944 PMC3728647 21508958

[43] Chong-Nguyen C, Yilmaz B, Coles B, Sokol H, MacPherson A, Siepe M, Reineke D, Mosbahi S, Tomii D, Nakase M, Atighetchi S, Ferro C, Wingert C, Gräni C, Pilgrim T, Windecker S, Blasco H, Dupuy C, Emond P, Siontis GCM. A scoping review evaluating the current state of gut microbiota and its metabolites in valvular heart disease physiopathology. Eur J Clin Invest. 2025;55(6):e14381. 10.1111/eci.14381. 39797472 10.1111/eci.14381

[44] Sarkar A, Yoo JY, Ozorio Dutra V, Morgan S, K. H., Groer M. The Association between Early-Life Gut Microbiota and Long-Term Health and Diseases. J Clin Med. 2021;10(3). 10.3390/jcm10030459. 10.3390/jcm10030459 PMC7865818 33504109

[45] Radua J. PRISMA 2020—An updated checklist for systematic reviews and meta-analyses. Neurosci Biobehav Rev. 2021;124:324–5. 10.1016/j.neubiorev.2021.02.016. 33596413 10.1016/j.neubiorev.2021.02.016

[46] Ding T, Schloss PD. Dynamics and associations of microbial community types across the human body. Nature. 2014;509(7500):357–60. 10.1038/nature13178. 24739969 10.1038/nature13178 PMC4139711

[47] Sinha T, Vich Vila A, Garmaeva S, Jankipersadsing SA, Imhann F, Collij V, Bonder MJ, Jiang X, Gurry T, Alm EJ, D’Amato M, Weersma RK, Scherjon S, Wijmenga C, Fu J, Kurilshikov A, Zhernakova A. Analysis of 1135 gut metagenomes identifies sex-specific resistome profiles. Gut Microbes. 2019;10(3):358–66. 10.1080/19490976.2018.1528822. 30373468 10.1080/19490976.2018.1528822 PMC6546312

[48] Lauretta R, Sansone M, Sansone A, Romanelli F, Appetecchia M. (2018). Gender in Endocrine Diseases: Role of Sex Gonadal Hormones. *International Journal of Endocrinology*, *2018*, 4847376. 10.1155/2018/4847376 10.1155/2018/4847376 PMC6215564 30420884

[49] Chen KL, Madak-Erdogan Z. Estrogen and Microbiota Crosstalk: Should We Pay Attention? Trends Endocrinol Metab. 2016;27(11):752–5. 10.1016/j.tem.2016.08.001. 27553057 10.1016/j.tem.2016.08.001

[50] Simpson ER. Sources of estrogen and their importance. J Steroid Biochem Mol Biol. 2003;86(3–5):225–30. 10.1016/s0960-0760(03)00360-1. 14623515 10.1016/s0960-0760(03)00360-1

[51] Flores R, Shi J, Fuhrman B, Xu X, Veenstra TD, Gail MH, Gajer P, Ravel J, Goedert JJ. Fecal microbial determinants of fecal and systemic estrogens and estrogen metabolites: A cross-sectional study. J Translational Med. 2012;10:253. 10.1186/1479-5876-10-253. 10.1186/1479-5876-10-253 PMC3552825 23259758

[52] Nelson LR, Bulun SE. Estrogen production and action. J Am Acad Dermatol. 2001;45(3 Suppl):116–24. 10.1067/mjd.2001.117432. 10.1067/mjd.2001.117432 11511861

[53] Shin J-H, Park Y-H, Sim M, Kim S-A, Joung H, Shin D-M. Serum level of sex steroid hormone is associated with diversity and profiles of human gut microbiome. Res Microbiol. 2019;170(4–5):192–201. 10.1016/j.resmic.2019.03.003. 30940469 10.1016/j.resmic.2019.03.003

[54] Insenser M, Murri M, Del Campo R, Martínez-García MÁ, Fernández-Durán E, Escobar-Morreale HF. Gut Microbiota and the Polycystic Ovary Syndrome: Influence of Sex, Sex Hormones, and Obesity. J Clin Endocrinol Metab. 2018;103(7):2552–62. 10.1210/jc.2017-02799. 29897462 10.1210/jc.2017-02799

[55] Pérez CE. Fruit and vegetable consumption. Health Rep. 2002;13(3):23–31. 12743958

[56] Conterno L, Martinelli F, Tamburini M, Fava F, Mancini A, Sordo M, Pindo M, Martens S, Masuero D, Vrhovsek U, Dal Lago C, Ferrario G, Morandini M, Tuohy K. Measuring the impact of olive pomace enriched biscuits on the gut microbiota and its metabolic activity in mildly hypercholesterolaemic subjects. Eur J Nutr. 2019;58(1):63–81. 10.1007/s00394-017-1572-2. 29124388 10.1007/s00394-017-1572-2 PMC6424929

[57] Iorga A, Cunningham CM, Moazeni S, Ruffenach G, Umar S, Eghbali M. The protective role of estrogen and estrogen receptors in cardiovascular disease and the controversial use of estrogen therapy. Biology Sex Differences. 2017;8(1):33. 10.1186/s13293-017-0152-8. 10.1186/s13293-017-0152-8 PMC5655818 29065927

[58] Li J, Zeng Q, Xiong Z, Xian G, Liu Z, Zhan Q, Lai W, Ao L, Meng X, Ren H, Xu D. Trimethylamine N-oxide induces osteogenic responses in human aortic valve interstitial cells in vitro and aggravates aortic valve lesions in mice. Cardiovascular Res. 2022;118(8):2018–30. 10.1093/cvr/cvab243. 10.1093/cvr/cvab243 34352088

[59] Xiong Z, Li J, Huang R, Zhou H, Xu X, Zhang S, Xie P, Li M, Guo Y, Liao X, Zhuang X. The gut microbe-derived metabolite trimethylamine-N-oxide induces aortic valve fibrosis via PERK/ATF-4 and IRE-1α/XBP-1s signaling in vitro and in vivo. Atherosclerosis. 2024;391:117431. 10.1016/j.atherosclerosis.2023.117431. 38408412 10.1016/j.atherosclerosis.2023.117431

[60] Almer G, Enko D, Kartiosuo N, Niinikoski H, Lehtimäki T, Munukka E, Viikari J, Rönnemaa T, Rovio SP, Mykkänen J, Lagström H, Jula A, Herrmann M, Raitakari OT, Meinitzer A, Pahkala K. Association of Serum Trimethylamine-N-Oxide Concentration from Childhood to Early Adulthood with Age and Sex. Clin Chem. 2024;70(9):1162–71. 10.1093/clinchem/hvae087. 38906833 10.1093/clinchem/hvae087

[61] Andraos S, Lange K, Clifford SA, Jones B, Thorstensen EB, Kerr JA, Wake M, Saffery R, Burgner DP, O’Sullivan JM. Plasma Trimethylamine N-Oxide and Its Precursors: Population Epidemiology, Parent-Child Concordance, and Associations with Reported Dietary Intake in 11- to 12-Year-Old Children and Their Parents. Curr Developments Nutr. 2020;4(7):nzaa103. 10.1093/cdn/nzaa103. 10.1093/cdn/nzaa103 PMC7335361 32666035

[62] Parra-Izquierdo I, Castaños-Mollor I, López J, Gómez C, San Román JA, Sánchez Crespo M, García-Rodríguez C. Lipopolysaccharide and interferon-γ team up to activate HIF-1α via STAT1 in normoxia and exhibit sex differences in human aortic valve interstitial cells. Biochim Et Biophys Acta Mol Basis Disease. 2019;1865(9):2168–79. 10.1016/j.bbadis.2019.04.014. 10.1016/j.bbadis.2019.04.014 31034990

[63] Parra-Izquierdo I, Castaños-Mollor I, López J, Gómez C, San Román JA, Sánchez Crespo M, García-Rodríguez C. Calcification Induced by Type I Interferon in Human Aortic Valve Interstitial Cells Is Larger in Males and Blunted by a Janus Kinase Inhibitor. Arterioscler Thromb Vasc Biol. 2018;38(9):2148–59. 10.1161/ATVBAHA.118.311504. 30026273 10.1161/ATVBAHA.118.311504

[64] Wen L, Lin X, Hu D, Li J, Xie K, Li S, Su S, Duan X, Zhong G, Lin Y, Chen Y, Xu T, Zeng Q. Trimethylamine N-oxide aggravates human aortic valve interstitial cell inflammation by regulating the macrophages polarization through a N6-methyladenosine-mediated pathway. Atherosclerosis. 2025;402:119109. 10.1016/j.atherosclerosis.2025.119109. 39952076 10.1016/j.atherosclerosis.2025.119109

[65] Liu Z, Li J, Liu H, Tang Y, Zhan Q, Lai W, Ao L, Meng X, Ren H, Xu D, Zeng Q. The intestinal microbiota associated with cardiac valve calcification differs from that of coronary artery disease. Atherosclerosis. 2019;284:121–8. 10.1016/j.atherosclerosis.2018.11.038. 30897381 10.1016/j.atherosclerosis.2018.11.038

[66] Naghipour S, Cox AJ, Fisher JJ, Plan M, Stark T, West N, Peart JN, Headrick JP, Du Toit EF. Circulating TMAO, the gut microbiome and cardiometabolic disease risk: An exploration in key precursor disorders. Diabetol Metab Syndr. 2024;16(1):133. 10.1186/s13098-024-01368-y. 38886825 10.1186/s13098-024-01368-y PMC11181661

[67] Sritharen Y, Enriquez-Sarano M, Schaff HV, Casaclang-Verzosa G, Miller JD. Pathophysiology of Aortic Valve Stenosis: Is It Both Fibrocalcific and Sex Specific? Physiol (Bethesda Md). 2017;32(3):182–96. 10.1152/physiol.00025.2016. 10.1152/physiol.00025.2016 PMC6148342 28404735

[68] Zhou Z, Sun L, Zhou W, Gao W, Yuan X, Zhou H, Ren Y, Li B, Wu Y, She J. Probiotic Bifidobacterium reduces serum TMAO in unstable angina patients via the gut to liver to heart axis. Liver Res. 2025;9(1):57–65. 10.1016/j.livres.2025.02.001. 40206430 10.1016/j.livres.2025.02.001 PMC11977283

[69] Delgado-Marin M, Sánchez-Esteban S, Cook-Calvete A, Jorquera-Ortega S, Zaragoza C, Saura M. Indoxyl Sulfate-Induced Valve Endothelial Cell Endothelial-to-Mesenchymal Transition and Calcification in an Integrin-Linked Kinase-Dependent Manner. Cells. 2024;13(6):481. 10.3390/cells13060481. 38534325 10.3390/cells13060481 PMC10969166

[70] Düsing P, Göbel I, Ackerschott A, Reese L, Giavalisco P, Dethloff F, Niepmann ST, Stei M, Beiert T, Zimmer S, Kurts C, Nickenig G, Jansen F, Zietzer A. The role of uremic toxin indoxyl sulfate in the pathophysiology of aortic valve stenosis. Cardiovascular Res. 2025;cvaf106. 10.1093/cvr/cvaf106. 10.1093/cvr/cvaf106 40570183

[71] Nakano, T., Katsuki, S., Chen, M., Decano, J. L., Halu, A., Lee, L. H., Pestana,D. V. S., Kum, A. S. T., Kuromoto, R. K., Golden, W. S., Boff, M. S., Guimaraes, G.C., Higashi, H., Kauffman, K. J., Maejima, T., Suzuki, T., Iwata, H., Barabási, A.-L.,Aster, J. C., … Aikawa, M. (2019). Uremic Toxin Indoxyl Sulfate Promotes Proinflammatory Macrophage Activation Via the Interplay of OATP2B1 and Dll4-Notch Signaling. *Circulation*, *139*(1), 78–96. 10.1161/CIRCULATIONAHA.118.034588. 10.1161/CIRCULATIONAHA.118.034588 PMC6311723 30586693

[72] Lee T-L, Hsuan C-F, Hsu C-C, Wei C-T, Wang C-P, Lu Y-C, Tang W-H, Lu N-H, Chung F-M, Lee Y-J, Tsai I-T. Associations of circulating total p-cresylsulfate and indoxyl sulfate concentrations with central obesity in patients with stable coronary artery disease: Sex-specific insights. Int J Obes. 2024;48(12):1775–84. 10.1038/s41366-024-01624-1. 10.1038/s41366-024-01624-1 PMC11584387 39237758

[73] Blanck HM, Gillespie C, Kimmons JE, Seymour JD, Serdula MK. Trends in fruit and vegetable consumption among U.S. men and women, 1994–2005. Prev Chronic Dis. 2008;5(2):A35. 18341771 PMC2396974

[74] Stea TH, Nordheim O, Bere E, Stornes P, Eikemo TA. Fruit and vegetable consumption in Europe according to gender, educational attainment and regional affiliation-A cross-sectional study in 21 European countries. PLoS ONE. 2020;15(5):e0232521. 10.1371/journal.pone.0232521. 32401798 10.1371/journal.pone.0232521 PMC7219700

[75] Pei J, Harakalova M, den Ruijter H, Pasterkamp G, Duncker DJ, Verhaar MC, Asselbergs FW, Cheng C. Cardiorenal disease connection during post-menopause: The protective role of estrogen in uremic toxins induced microvascular dysfunction. Int J Cardiol. 2017;238:22–30. 10.1016/j.ijcard.2017.03.050. 28341374 10.1016/j.ijcard.2017.03.050

[76] Le Nezet E, Marqueze-Pouey C, Guisle I, Clavel M-A. Molecular Features of Calcific Aortic Stenosis in Female and Male Patients. CJC Open. 2024;6(9):1125–37. 10.1016/j.cjco.2024.06.002. 39525825 10.1016/j.cjco.2024.06.002 PMC11544188

[77] Appleby C, Bleiziffer S, Bramlage P, Delgado V, Eltchaninoff H, Gebhard C, Hengstenberg C, Kurucova J, Marx P, Rudolph TK, Wojakowski W. Sex-related disparities in aortic stenosis from disease awareness to treatment: A state-of-the-art review. J Thorac Disease. 2024;16(9):6308–19. 10.21037/jtd-24-406. 39444914 10.21037/jtd-24-406 PMC11494558

[78] Springhetti P, Abdoun K, Clavel M-A. Sex Differences in Aortic Stenosis: From the Pathophysiology to the Intervention, Current Challenges, and Future Perspectives. J Clin Med. 2024;13(14):4237. 10.3390/jcm13144237. 39064275 10.3390/jcm13144237 PMC11278486

[79] Tribouilloy C, Bohbot Y, Rusinaru D, Belkhir K, Diouf M, Altes A, Delpierre Q, Serbout S, Kubala M, Levy F, Maréchaux S, Sarano E, M. Excess Mortality and Undertreatment of Women With Severe Aortic Stenosis. J Am Heart Association. 2021;10(1):e018816. 10.1161/JAHA.120.018816. 10.1161/JAHA.120.018816 PMC7955469 33372529

[80] Ito S, Miranda WR, Nkomo VT, Lewis BR, Oh JK. Sex Differences in LV Remodeling and Hemodynamics in Aortic Stenosis: Sex-Specific Criteria for Severe Stenosis? JACC Cardiovasc Imaging. 2022;15(7):1175–89. 10.1016/j.jcmg.2022.02.007. 35798393 10.1016/j.jcmg.2022.02.007

[81] Iribarren AC, AlBadri A, Wei J, Nelson MD, Li D, Makkar R, Merz CNB. Sex differences in aortic stenosis: Identification of knowledge gaps for sex-specific personalized medicine. Am Heart J Plus: Cardiol Res Pract. 2022;21:100197. 10.1016/j.ahjo.2022.100197. 10.1016/j.ahjo.2022.100197 PMC9629620 36330169

[82] Williams M, Kodali SK, Hahn RT, Humphries KH, Nkomo VT, Cohen DJ, Douglas PS, Mack M, McAndrew TC, Svensson L, Thourani VH, Tuzcu EM, Weissman NJ, Kirtane AJ, Leon MB. Sex-related differences in outcomes after transcatheter or surgical aortic valve replacement in patients with severe aortic stenosis: Insights from the PARTNER Trial (Placement of Aortic Transcatheter Valve). J Am Coll Cardiol. 2014;63(15):1522–8. 10.1016/j.jacc.2014.01.036. 24561149 10.1016/j.jacc.2014.01.036

[83] Otto CM. Calcific aortic stenosis—Time to look more closely at the valve. N Engl J Med. 2008;359(13):1395–8. 10.1056/NEJMe0807001. 18815402 10.1056/NEJMe0807001

[84] Pawade T, Sheth T, Guzzetti E, Dweck MR, Clavel M-A. Why and How to Measure Aortic Valve Calcification in Patients With Aortic Stenosis. JACC Cardiovasc Imaging. 2019;12(9):1835–48. 10.1016/j.jcmg.2019.01.045. 31488252 10.1016/j.jcmg.2019.01.045

[85] Côté N, Clavel M-A. Sex Differences in the Pathophysiology, Diagnosis, and Management of Aortic Stenosis. Cardiol Clin. 2020;38(1):129–38. 10.1016/j.ccl.2019.09.008. 31753171 10.1016/j.ccl.2019.09.008

[86] Gać P, Jaworski A, Grajnert F, Kicman K, Trejtowicz-Sutor A, Witkowski K, Poręba M, Poręba R. Aortic Valve Calcium Score: Applications in Clinical Practice and Scientific Research-A Narrative Review. J Clin Med. 2024;13(14):4064. 10.3390/jcm13144064. 39064103 10.3390/jcm13144064 PMC11277735

[87] Aguado BA, Walker CJ, Grim JC, Schroeder ME, Batan D, Vogt BJ, Rodriguez AG, Schwisow JA, Moulton KS, Weiss RM, Heistad DD, Leinwand LA, Anseth KS. Genes That Escape X Chromosome Inactivation Modulate Sex Differences in Valve Myofibroblasts. Circulation. 2022;145(7):513–30. 10.1161/CIRCULATIONAHA.121.054108. 35000411 10.1161/CIRCULATIONAHA.121.054108 PMC8844107

[88] Voisine M, Hervault M, Shen M, Boilard A-J, Filion B, Rosa M, Bossé Y, Mathieu P, Côté N, Clavel M-A. Age, Sex, and Valve Phenotype Differences in Fibro-Calcific Remodeling of Calcified Aortic Valve. J Am Heart Association. 2020;9(10):e015610. 10.1161/JAHA.119.015610. 10.1161/JAHA.119.015610 PMC7660864 32384012

[89] Patel PP, Sabbagh E, Johnson A, Suliman PW, Salwa R, Morales-Lara N, Pollak AC, Yamani P, Parikh M, Sonavane P, Landolfo SK, Alkhouli C, Eleid MA, Guerrero MF, Fortuin M, Sweeney FD, Noseworthy J, Carter PA, R. E., Adedinsewo D. Sex Differences in the Impact of Aortic Valve Calcium Score on Mortality After Transcatheter Aortic Valve Replacement. Circ Cardiovasc Imaging. 2022;15(8):e014034. 10.1161/CIRCIMAGING.122.014034. 35920157 10.1161/CIRCIMAGING.122.014034 PMC9397521

[90] Tastet L, Kwiecinski J, Pibarot P, Capoulade R, Everett RJ, Newby DE, Shen M, Guzzetti E, Arsenault M, Bédard É, Larose É, Beaudoin J, Dweck M, Clavel M-A. Sex-Related Differences in the Extent of Myocardial Fibrosis in Patients With Aortic Valve Stenosis. JACC Cardiovasc Imaging. 2020;13(3):699–711. 10.1016/j.jcmg.2019.06.014. 31422128 10.1016/j.jcmg.2019.06.014

[91] Bienjonetti-Boudreau D, Fleury M-A, Voisine M, Paquin A, Chouinard I, Tailleur M, Duval R, Magnan P-O, Beaudoin J, Salaun E, Clavel M-A. Impact of sex on the management and outcome of aortic stenosis patients. Eur Heart J. 2021;42(27):2683–91. 10.1093/eurheartj/ehab242. 34023890 10.1093/eurheartj/ehab242

[92] Guo Y, Xu S, Zhan H, Chen H, Hu P, Zhou D, Dai H, Liu X, Hu W, Zhu G, Suzuki T, Wang J. Trimethylamine N-Oxide Levels Are Associated with Severe Aortic Stenosis and Predict Long-Term Adverse Outcome. J Clin Med. 2023;12(2):407. 10.3390/jcm12020407. 36675336 10.3390/jcm12020407 PMC9861904

[93] Mo C, Lou X, Xue J, Shi Z, Zhao Y, Wang F, Chen G. The influence of Akkermansia muciniphila on intestinal barrier function. Gut Pathogens. 2024;16:41. 10.1186/s13099-024-00635-7. 39097746 10.1186/s13099-024-00635-7 PMC11297771

[94] Duranti S, Ruiz L, Lugli GA, Tames H, Milani C, Mancabelli L, Mancino W, Longhi G, Carnevali L, Sgoifo A, Margolles A, Ventura M, Ruas-Madiedo P, Turroni F. Bifidobacterium adolescentis as a key member of the human gut microbiota in the production of GABA. Sci Rep. 2020;10(1):14112. 10.1038/s41598-020-70986-z. 32839473 10.1038/s41598-020-70986-z PMC7445748

[95] Derrien M, Turroni F, Ventura M, van Sinderen D. Insights into endogenous Bifidobacterium species in the human gut microbiota during adulthood. Trends Microbiol. 2022;30(10):940–7. 10.1016/j.tim.2022.04.004. 35577716 10.1016/j.tim.2022.04.004

[96] Nelson V, Patil V, Simon LR, Schmidt K, McCoy CM, Masters KS. Angiogenic Secretion Profile of Valvular Interstitial Cells Varies With Cellular Sex and Phenotype. Front Cardiovasc Med. 2021;8:736303. 10.3389/fcvm.2021.736303. 34527715 10.3389/fcvm.2021.736303 PMC8435671

[97] Le Nezet E, Marqueze-Pouey C, Guisle I, Clavel M-A. Molecular Features of Calcific Aortic Stenosis in Female and Male Patients. CJC Open. 2024;6(9):1125–37. 10.1016/j.cjco.2024.06.002. 39525825 10.1016/j.cjco.2024.06.002 PMC11544188

[98] Masjedi S, Lei Y, Patel J, Ferdous Z. Sex-related differences in matrix remodeling and early osteogenic markers in aortic valvular interstitial cells. Heart Vessels. 2017;32(2):217–28. 10.1007/s00380-016-0909-8. 27761653 10.1007/s00380-016-0909-8

[99] Nelson V, Patil V, Simon LR, Schmidt K, McCoy CM, Masters KS. Angiogenic Secretion Profile of Valvular Interstitial Cells Varies With Cellular Sex and Phenotype. Front Cardiovasc Med. 2021;8:736303. 10.3389/fcvm.2021.736303. 34527715 10.3389/fcvm.2021.736303 PMC8435671

[100] Matilla L, Martín-Núñez E, Garaikoetxea M, Navarro A, Vico JA, Arrieta V, García-Peña A, Fernández-Celis A, Gainza A, Álvarez V, Sádaba R, López-Andrés N, Jover E. Characterization of the sex-specific pattern of angiogenesis and lymphangiogenesis in aortic stenosis. Front Cardiovasc Med. 2022;9:971802. 10.3389/fcvm.2022.971802. 36172587 10.3389/fcvm.2022.971802 PMC9510663

[101] Myasoedova VA, Massaiu I, Moschetta D, Chiesa M, Songia P, Valerio V, Alfieri V, Capoulade R, Trabattoni D, Andreini D, Mass E, Parisi V, Poggio P. Sex-Specific Cell Types and Molecular Pathways Indicate Fibro-Calcific Aortic Valve Stenosis. Front Immunol. 2022;13:747714. 10.3389/fimmu.2022.747714. 35280999 10.3389/fimmu.2022.747714 PMC8907138

[102] Parra-Izquierdo I, Castaños-Mollor I, López J, Gómez C, San Román JA, Sánchez Crespo M, García-Rodríguez C. Calcification Induced by Type I Interferon in Human Aortic Valve Interstitial Cells Is Larger in Males and Blunted by a Janus Kinase Inhibitor. Arterioscler Thromb Vasc Biol. 2018;38(9):2148–59. 10.1161/ATVBAHA.118.311504. 30026273 10.1161/ATVBAHA.118.311504

[103] Prabutzki P, Wölk M, Böttner J, Ni Z, Werner S, Thiele H, Schiller J, Büttner P, Schlotter F, Fedorova M. Sex-specific lipidomic signatures in aortic valve disease reflect differential fibro-calcific progression. Nat Commun. 2025;16(1):5163. 10.1038/s41467-025-60411-2. 40461477 10.1038/s41467-025-60411-2 PMC12134101

[104] Le Nezet E, Marqueze-Pouey C, Guisle I, Clavel M-A. Molecular Features of Calcific Aortic Stenosis in Female and Male Patients. CJC Open. 2024;6(9):1125–37. 10.1016/j.cjco.2024.06.002. 39525825 10.1016/j.cjco.2024.06.002 PMC11544188

[105] Matilla L, Jover E, Garaikoetxea M, Martín-Nuñez E, Arrieta V, García-Peña A, Navarro A, Fernández-Celis A, Gainza A, Álvarez V, Álvarez, de la Rosa D, Sádaba R, Jaisser F, López-Andrés N. (2022). Sex-Related Signaling of Aldosterone/Mineralocorticoid Receptor Pathway in Calcific Aortic Stenosis. *Hypertension*, *79*(8), 1724–1737. 10.1161/HYPERTENSIONAHA.122.19526 10.1161/HYPERTENSIONAHA.122.19526 35549329

[106] Garg V, Muth AN, Ransom JF, Schluterman MK, Barnes R, King IN, Grossfeld PD, Srivastava D. Mutations in NOTCH1 cause aortic valve disease. Nature. 2005;437(7056):270–4. 10.1038/nature03940. 16025100 10.1038/nature03940

[107] Balaji S, Dong X, Li H, Zhang Y, Steen E, Lingappan K. Sex-specific differences in primary neonatal murine lung fibroblasts exposed to hyperoxia in vitro: Implications for bronchopulmonary dysplasia. Physiol Genom. 2018;50(11):940–6. 10.1152/physiolgenomics.00075.2018. 10.1152/physiolgenomics.00075.2018 PMC6293119 30169132

[108] Le Nezet E, Marqueze-Pouey C, Guisle I, Clavel M-A. Molecular Features of Calcific Aortic Stenosis in Female and Male Patients. CJC Open. 2024;6(9):1125–37. 10.1016/j.cjco.2024.06.002. 39525825 10.1016/j.cjco.2024.06.002 PMC11544188

[109] Shah TA, Rogers MB. Unanswered Questions Regarding Sex and BMP/TGF-β Signaling. J Dev Biology. 2018;6(2):14. 10.3390/jdb6020014. 10.3390/jdb6020014 PMC6027345 29914150

[110] Khan K, Yu B, Kiwan C, Shalal Y, Filimon S, Cipro M, Shum-Tim D, Cecere R, Schwertani A. The Role of Wnt/β-Catenin Pathway Mediators in Aortic Valve Stenosis. Front Cell Dev Biology. 2020;8:862. 10.3389/fcell.2020.00862. 10.3389/fcell.2020.00862 PMC7513845 33015048

[111] Bhat M, Pasini E, Pastrello C, Angeli M, Baciu C, Abovsky M, Coffee A, Adeyi O, Kotlyar M, Jurisica I. Estrogen Receptor 1 Inhibition of Wnt/β-Catenin Signaling Contributes to Sex Differences in Hepatocarcinogenesis. Front Oncol. 2021;11:777834. 10.3389/fonc.2021.777834. 34881186 10.3389/fonc.2021.777834 PMC8645636

[112] Matilla L, Jover E, Garaikoetxea M, Martín-Nuñez E, Arrieta V, García-Peña A, Navarro A, Fernández-Celis A, Gainza A, Álvarez V, Álvarez, de la Rosa D, Sádaba R, Jaisser F, López-Andrés N. (2022). Sex-Related Signaling of Aldosterone/Mineralocorticoid Receptor Pathway in Calcific Aortic Stenosis. *Hypertension (Dallas, Tex.: 1979)*, *79*(8), 1724–1737. 10.1161/HYPERTENSIONAHA.122.19526 10.1161/HYPERTENSIONAHA.122.19526 35549329

[113] Chakrabarti S, Lekontseva O, Davidge ST. Estrogen is a modulator of vascular inflammation. IUBMB Life. 2008;60(6):376–82. 10.1002/iub.48. 18409173 10.1002/iub.48

[114] Simon LR, Scott AJ, Rios F, Zembles L, J., Masters KS. Cellular-scale sex differences in extracellular matrix remodeling by valvular interstitial cells. Heart Vessels. 2023;38(1):122–30. 10.1007/s00380-022-02164-2. 36070095 10.1007/s00380-022-02164-2 PMC10120251

[115] Mahmoodzadeh S, Dworatzek E, Fritschka S, Pham TH, Regitz-Zagrosek V. 17beta-Estradiol inhibits matrix metalloproteinase-2 transcription via MAP kinase in fibroblasts. Cardiovascular Res. 2010;85(4):719–28. 10.1093/cvr/cvp350. 10.1093/cvr/cvp350 PMC2819834 19861308

[116] Yanes LL, Romero DG, Iles JW, Iliescu R, Gomez-Sanchez C, Reckelhoff JF. Sexual dimorphism in the renin-angiotensin system in aging spontaneously hypertensive rats. Am J Physiol Regul Integr Comp Physiol. 2006;291(2):R383–390. 10.1152/ajpregu.00510.2005. 16914423 10.1152/ajpregu.00510.2005

[117] Matilla L, Garaikoetxea M, Arrieta V, García-Peña A, Fernández-Celis A, Navarro A, Gainza A, Álvarez V, Sádaba R, Jover E, López-Andrés N. Sex-Differences in Aortic Stenosis: Mechanistic Insights and Clinical Implications. Front Cardiovasc Med. 2022;9:818371. 10.3389/fcvm.2022.818371. 35282345 10.3389/fcvm.2022.818371 PMC8907577

[118] Osako MK, Nakagami H, Koibuchi N, Shimizu H, Nakagami F, Koriyama H, Shimamura M, Miyake T, Rakugi H, Morishita R. Estrogen inhibits vascular calcification via vascular RANKL system: Common mechanism of osteoporosis and vascular calcification. Circul Res. 2010;107(4):466–75. 10.1161/CIRCRESAHA.110.216846. 10.1161/CIRCRESAHA.110.216846 20595654

[119] Baker JM, Al-Nakkash L, Herbst-Kralovetz MM. Estrogen-gut microbiome axis: Physiological and clinical implications. Maturitas. 2017;103:45–53. 10.1016/j.maturitas.2017.06.025. 28778332 10.1016/j.maturitas.2017.06.025

[120] Zhu D, Hadoke PWF, Wu J, Vesey AT, Lerman DA, Dweck MR, Newby DE, Smith LB, MacRae VE. Ablation of the androgen receptor from vascular smooth muscle cells demonstrates a role for testosterone in vascular calcification. Sci Rep. 2016;6:24807. 10.1038/srep24807. 27095121 10.1038/srep24807 PMC4837411

[121] Eildermann K, Goldmann S, Krause U, Backhoff D, Schöndube FA, Paul T, Quentin T, Müller MJ. Differences in Androgen Receptor Expression in Human Heart Tissue in Various Types of Cardiomyopathy and in Aortic Valve Stenosis. J Cardiovasc Dev Disease. 2023;10(11):466. 10.3390/jcdd10110466. 37998524 10.3390/jcdd10110466 PMC10672689

[122] Pawade, T., Clavel, M.-A., Tribouilloy, C., Dreyfus, J., Mathieu, T., Tastet, L.,Renard, C., Gun, M., Jenkins, W. S. A., Macron, L., Sechrist, J. W., Lacomis, J. M.,Nguyen, V., Galian Gay, L., Cuéllar Calabria, H., Ntalas, I., Cartlidge, T. R. G.,Prendergast, B., Rajani, R., … Dweck, M. R. (2018). Computed Tomography Aortic Valve Calcium Scoring in Patients With Aortic Stenosis. *Circulation. Cardiovascular Imaging*, *11*(3), e007146. 10.1161/CIRCIMAGING.117.007146. 10.1161/CIRCIMAGING.117.007146 29555836

[123] Candellier A, Issa N, Grissi M, Brouette T, Avondo C, Gomila C, Blot G, Gubler B, Touati G, Bennis Y, Caus T, Brazier M, Choukroun G, Tribouilloy C, Kamel S, Boudot C, Hénaut L. Indoxyl-sulfate activation of the AhR- NF-κB pathway promotes interleukin-6 secretion and the subsequent osteogenic differentiation of human valvular interstitial cells from the aortic valve. J Mol Cell Cardiol. 2023;179:18–29. 10.1016/j.yjmcc.2023.03.011. & Stop-As Investigators 36967106 10.1016/j.yjmcc.2023.03.011

[124] Zhang Y, Chen R, Zhang D, Qi S, Liu Y. Metabolite interactions between host and microbiota during health and disease: Which feeds the other? Biomed Pharmacother. 2023;160:114295. 10.1016/j.biopha.2023.114295. 36709600 10.1016/j.biopha.2023.114295

[125] *Ruminococcus—An overview | ScienceDirect Topics*. (o. J.). Abgerufen 28. August 2025, von https://www.sciencedirect.com/topics/biochemistry-genetics-and-molecular-biology/ruminococcus

[126] Dempsey E, Corr SC. Lactobacillus spp. for Gastrointestinal Health: Current and Future Perspectives. Front Immunol. 2022;13:840245. 10.3389/fimmu.2022.840245. 35464397 10.3389/fimmu.2022.840245 PMC9019120

[127] Fleury M-A, Annabi M-S, Voisine M, Hervault M, Boilard A-J, Shen M, Marette A, Côté N, Clavel M-A. Impact of sex and sex hormones on pathophysiology and progression of aortic stenosis in a murine model. Physiological Rep. 2022;10(16):e15433. 10.14814/phy2.15433. 10.14814/phy2.15433 PMC9419154 36029186

[128] Joll JE, Bersi MR, Nyman JS, Merryman WD. Evaluation of early bilateral ovariectomy in mice as a model of left heart disease. Am J Physiol Heart Circ Physiol. 2022;322(6):H1080–5. 10.1152/ajpheart.00157.2022. 35486477 10.1152/ajpheart.00157.2022 PMC9142153

[129] Billig H, Schmitt J, Singer L, Bourauel C, Schildberg FA, Masson W, Lübbering N, Vogel W, Perner S, Stei M, Niepmann S, Silaschi M, Bakhtiary F, Nickenig G, Zimmer S. Aortic valve stenosis and osteoporosis: Insights from a mouse model. BMC Cardiovasc Disord. 2025;25(1):562. 10.1186/s12872-025-05037-4. 40745526 10.1186/s12872-025-05037-4 PMC12312273

[130] Fliegner D, Ellieva A, Angelov A, Petrov G, Regitz-Zagrosek V. Sex differences and estrogen effects in cardiac mitochondria in human aortic stenosis and in the mouse heart. Front Endocrinol. 2023;14:1181044. 10.3389/fendo.2023.1181044. 10.3389/fendo.2023.1181044 PMC10617023 37916152

[131] Al Akhdar J, Yangın Yılmaz MN, Baysal K. TMAO-Triggered Endothelial-Mesenchymal Transition and Microvesicle Release as Mediators of Vascular Smooth Muscle Cell Osteogenic Differentiation and Vascular Calcification. Cells. 2026;15(5):466. 10.3390/cells15050466. 41827899 10.3390/cells15050466 PMC12984406

[132] Lei Y, Xiong G, Lei D, Wu H, Peng X, Chen L, Fang Q, Wu Y, Wu Y, Li X, Li Y. Gut microbiota-derived TMAO drives the kidney-bone-vascular axis in chronic kidney disease complications. Ren Fail. 2025;47(1):2575434. 10.1080/0886022X.2025.2575434. 41232933 10.1080/0886022X.2025.2575434 PMC12616656

[133] Yadgir, S., Johnson, C. O., Aboyans, V., Adebayo, O. M., Adedoyin, R. A., Afarideh,M., Alahdab, F., Alashi, A., Alipour, V., Arabloo, J., Azari, S., Barthelemy, C. M.,Benziger, C. P., Berman, A. E., Bijani, A., Carrero, J. J., Carvalho, F., Daryani,A., Durães, A. R., … Global Burden of Disease Study 2017 Nonrheumatic Valve Disease Collaborators. (2020). Global, Regional, and National Burden of Calcific Aortic Valve and Degenerative Mitral Valve Diseases, 1990–2017. *Circulation*, *141*(21), 1670–1680. 10.1161/CIRCULATIONAHA.119.043391. 10.1161/CIRCULATIONAHA.119.043391 32223336

[134] Nam EH, Lee M, Kim D, Jung YH, Yang J, Shin M. Folate Production by Streptococcus thermophilus IDCC 2201 and Its Impact. Hum Gut Microbiota. 2025;35(5):1–13. 10.4014/jmb.2502.02045. 10.4014/jmb.2502.02045 PMC12149402 40443239

[135] Cho G-S, Ritzmann F, Eckstein M, Huch M, Briviba K, Behsnilian D, Neve H, Franz CMAP. Quantification of Slackia and Eggerthella spp. In Human Feces and Adhesion of Representatives Strains to Caco-2 Cells. Front Microbiol. 2016;7. 10.3389/fmicb.2016.00658. 10.3389/fmicb.2016.00658 PMC4860493 27242689

[136] Gao X, Mu P, Zhu X, Chen X, Tang S, Wu Y, Miao X, Wang X, Wen J, Deng Y. Dual Function of a Novel Bacterium, Slackia sp. D-G6: Detoxifying Deoxynivalenol and Producing the Natural Estrogen Analogue, Equol. Toxins. 2020;12(2):85. 10.3390/toxins12020085. 31991913 10.3390/toxins12020085 PMC7076803

[137] Bode LM, Bunzel D, Huch M, Cho G-S, Ruhland D, Bunzel M, Bub A, Franz CM, Kulling SE. In vivo and in vitro metabolism of *trans*-resveratrol by human gut microbiota123. Am J Clin Nutr. 2013;97(2):295–309. 10.3945/ajcn.112.049379. 23283496 10.3945/ajcn.112.049379

[138] Thomas F, Hehemann J-H, Rebuffet E, Czjzek M, Michel G. Environmental and Gut Bacteroidetes: The Food Connection. Front Microbiol. 2011;2:93. 10.3389/fmicb.2011.00093. 21747801 10.3389/fmicb.2011.00093 PMC3129010

[139] Johnson EL, Heaver SL, Walters WA, Ley RE. Microbiome and metabolic disease: Revisiting the bacterial phylum Bacteroidetes. J Mol Med. 2017;95(1):1–8. 10.1007/s00109-016-1492-2. 27900395 10.1007/s00109-016-1492-2 PMC5187364

[140] Yeoh YK, Sun Y, Ip LYT, Wang L, Chan FKL, Miao Y, Ng SC. Prevotella species in the human gut is primarily comprised of Prevotella copri, Prevotella stercorea and related lineages. Sci Rep. 2022;12(1):9055. 10.1038/s41598-022-12721-4. 35641510 10.1038/s41598-022-12721-4 PMC9156738

[141] Precup G, Vodnar D-C. Gut Prevotella as a possible biomarker of diet and its eubiotic versus dysbiotic roles: A comprehensive literature review. Br J Nutr. 2019;122(2):131–40. 10.1017/S0007114519000680. 30924428 10.1017/S0007114519000680

[142] Zafar H, Saier MH. Gut Bacteroides species in health and disease. Gut Microbes. o. J.;13(1):1848158. 10.1080/19490976.2020.1848158. 10.1080/19490976.2020.1848158 PMC7872030 33535896

[143] Zhang S-M, Huang S-L. The Commensal Anaerobe Veillonella dispar Reprograms Its Lactate Metabolism and Short-Chain Fatty Acid Production during the Stationary Phase. Microbiol Spectr. 2023;11(2):e0355822. 10.1128/spectrum.03558-22. 36975840 10.1128/spectrum.03558-22 PMC10100942

[144] Malat I, Drancourt M, Grine G. Methanobrevibacter smithii cell variants in human physiology and pathology: A review. Heliyon. 2024;10(18):e36742. 10.1016/j.heliyon.2024.e36742. 39347381 10.1016/j.heliyon.2024.e36742 PMC11437934

[145] Duller S, Vrbancic S, Szydłowski Ł, Mahnert A, Blohs M, Predl M, Kumpitsch C, Zrim V, Högenauer C, Kosciolek T, Schmitz RA, Eberhard A, Dragovan M, Schmidberger L, Zurabischvili T, Weinberger V, Moser AM, Kolb D, Pernitsch D, Moissl-Eichinger C. Targeted isolation of Methanobrevibacter strains from fecal samples expands the cultivated human archaeome. Nat Commun. 2024;15(1):7593. 10.1038/s41467-024-52037-7. 39217206 10.1038/s41467-024-52037-7 PMC11366006

[146] Racherla J. (2023, Dezember 13). *Coprococcus in your gut: The secret of happiness? | Cambridge Core Blog*. https://www.cambridge.org/core/blog/2023/12/13/coprococcus-in-your-gut-the-secret-of-happiness/

[147] Notting F, Pirovano W, Sybesma W, Kort R. The butyrate-producing and spore-forming bacterial genus Coprococcus as a potential biomarker for neurological disorders. Gut Microbiome. 2023;4:e16. 10.1017/gmb.2023.14. 39295905 10.1017/gmb.2023.14 PMC11406416

[148] Muleshkova T, Bazukyan I, Papadimitriou K, Gotcheva V, Angelov A, Dimov SG. Exploring the Multifaceted Genus Acinetobacter: The Facts, the Concerns and the Oppoptunities the Dualistic Geuns Acinetobacter. J Microbiol Biotechnol. 2025;35:e2411043. 10.4014/jmb.2411.11043. 40081886 10.4014/jmb.2411.11043 PMC11925754

[149] Companys J, Gosalbes MJ, Pla-Pagà L, Calderón-Pérez L, Llauradó E, Pedret A, Valls RM, Jiménez-Hernández N, Sandoval-Ramirez BA, del Bas JM, Caimari A, Rubió L, Solà R. Gut Microbiota Profile and Its Association with Clinical Variables and Dietary Intake in Overweight/Obese and Lean Subjects: A Cross-Sectional Study. Nutrients. 2021;13(6). 10.3390/nu13062032. Article 6. 10.3390/nu13062032 PMC8231825 34199239

[150] Wu, C., Yang, F., Zhong, H., Hong, J., Lin, H., Zong, M., Ren, H., Zhao, S., Chen,Y., Shi, Z., Wang, X., Shen, J., Wang, Q., Ni, M., Chen, B., Cai, Z., Zhang, M., Cao,Z., Wu, K., … Liu, R. (2024). Obesity-enriched gut microbe degrades myo-inositol and promotes lipid absorption. *Cell Host & Microbe*, *32*(8), 1301–1314.e9. 10.1016/j.chom.2024.06.012. 10.1016/j.chom.2024.06.012 38996548

[151] Yang X, Zhang M, Liu Y, Wei F, Li X, Feng Y, Jin X, Liu D, Guo Y, Hu Y. Inulin-enriched Megamonas funiformis ameliorates metabolic dysfunction-associated fatty liver disease by producing propionic acid. Npj Biofilms Microbiomes. 2023;9(1):84. 10.1038/s41522-023-00451-y. 37925493 10.1038/s41522-023-00451-y PMC10625582

[152] Nature RC. by S. (2022, November 4). *Keepers of the host: Paraprevotella spp. in the large intestine*. Research Communities by Springer Nature. http://microbiologycommunity.nature.com/posts/jkljkjklj

[153] Gu F, Zhu S, Tang Y, Liu X, Jia M, Malmuthuge N, Valencak TG, McFadden JW, Liu J-X, Sun H-Z. Gut microbiome is linked to functions of peripheral immune cells in transition cows during excessive lipolysis. Microbiome. 2023;11(1):40. 10.1186/s40168-023-01492-3. 36869370 10.1186/s40168-023-01492-3 PMC9983187

